# Diet and Physical Activity Interventions for People from Minority Ethnic Backgrounds in the UK: A Scoping Review Exploring Barriers, Enablers and Cultural Adaptations

**DOI:** 10.1007/s40615-024-02112-y

**Published:** 2024-08-15

**Authors:** Thando Katangwe-Chigamba, Kumud Kantilal, Joseph Hartley-Palmer, Shukrat O. Salisu-Olatunji, Carys Seeley, Felix Naughton, Rachel Chester

**Affiliations:** 1 Norwich Clinical Trials Unit, Norwich Medical School, University of East Anglia, Norwich, United Kingdom; 2 Research Department of Primary Care & Population Health, University College London, London, United Kingdom; 3 School of Health Sciences, Faculty of Medicine and Health, University of East Anglia, Norwich, United Kingdom; 4 Department of Population Health Sciences, University of Leicester, Leicester, United Kingdom

**Keywords:** Ethnic minority, Diabetes Prevention; Cultural adaptations, Tailored interventions, Lifestyle interventions

## Abstract

**Background:**

Type 2 diabetes (T2D) and cardiovascular disease (CVD) are a global pandemic, driven by obesity, poor diet and physical inactivity. In the UK, the prevalence of T2D and CVD is higher in minority ethnic groups. Lifestyle prevention interventions can be effective but uptake amongst minority ethnic groups in the UK is low and the extent of cultural adaptations to increase engagement unknown.

**Aim:**

To explore barriers, enablers and culturally adapted lifestyle interventions in UK minority ethnic groups.

**Methods:**

Four electronic databases were searched from to January 2013–2023. Two independent reviewers carried out manuscript selection and data extraction. Barriers and enablers were mapped to the Capability + Opportunity + Motivation = Behaviour (COM-B) theoretical model. Intervention adaptations were linked to behaviour change strategies and reported within a Cultural Adaptation framework.

**Results:**

Twenty-three studies were included, reporting barriers/enablers, culturally adapted interventions or both. Barriers and enablers mostly mapped to social and physical opportunity, and reflective motivation. Common adaptation strategies considered behavioural influences related to culture, values, religious beliefs and/or traditions. Most impactful strategies were associated with using credible sources of information and reorganising social and environmental contexts.

**Discussion and conclusions:**

The current umbrella approach to preventative intervention delivery is unlikely to promote sustained participation in behaviour change amongst UK ethnic minorities. Engagement strategies for this population should consider key determinants such as social contexts, beliefs and cultural norms. Important research gaps include interventions investigating tailored interventions for Black populations, and the impact of negative social experiences (e.g., racism) on engagement.

## Background

The global prevalence of Type 2 diabetes (T2D) has increased rapidly over recent decades [1, 2], with 693 million adults predicted to have diabetes by 2045 [3]. In the United Kingdom (UK), it is estimated that 5.5 million people will be living with diabetes by 2030 [4]. The management of diabetes and its complications costs the UK National Health Service (NHS) approximately 10% of the total budget [5, 6].

The prevalence of T2D is higher among minority ethnic groups in the UK[7, 8]. People of Asian, Black African and Caribbean ethnicities are two-to-four times more likely to have diabetes, develop T2D at lower weight thresholds, and are diagnosed 10–12 years earlier than people of White ethnicities [7, 9]. The risk of developing T2D depends on multiple non-modifiable and modifiable risk factors including age, family history, ethnicity, socioeconomic status, and being overweight or obese [10]. Obesity accounts for approximately 80–85% of the overall risk of developing T2D and together with physical inactivity, is estimated to cause a large proportion of the global diabetes burden [11]. In England, obesity affects 25.9% of the adult population, with the highest prevalence amongst those who identified as Black British (33.7%)[12]. Other factors that can predispose minority ethnic groups to a higher risk of developing T2D include a higher genetic predisposition and enhanced susceptibility for cardio-metabolic complications in relationship to body composition [9, 13–16].

Minority ethnic groups in high-income countries such as the UK also suffer disproportionately from diabetes-related complications such as cardiovascular disease (CVD) [17]. CVD is the most prevalent cause of morbidity and mortality in people with diabetes, affecting almost a third of people with T2D[18, 19].

The prevention of T2D and related conditions (e.g. CVD) through early detection, lifestyle changes and obesity prevention are amongst the key priorities outlined in the NHS long term plan [20]. In England, the NHS Diabetes Prevention Programme (NHS DPP) and the NHS Health Check are two main interventions designed to prevent T2D and CVD respectively. The NHS DPP, introduced in 2016, identifies people at high risk of diabetes and refers them to a 9-month behavioural change intervention primarily consisting of diabetes and lifestyle education and support to adopt a healthy diet, increase physical activity and reduce weight [21]. The effectiveness of DPPs in delaying or preventing the incidence of T2D has been established by several randomised controlled trials conducted in Finland, India, US, China and Australia [22–29]. A recent evaluation of the NHS DPP, which included a predominantly White population (~ 84%), has demonstrated effectiveness at reducing the incidence of T2D in people with prediabetes [30]. However, NHS DPP outcome reports indicate that minority ethnic groups including Black and Asian populations are 25% less likely to complete the programme and have smaller HbA1c and weight reductions[31–33]. The NHS Health Check, a CVD prevention programme introduced in 2009, is freely offered to adults aged 40–74 years every 5 years and encompasses a risk assessment, risk communication (risk of developing heart disease, stroke, T2D or kidney disease, over the next 10 years) and risk management through tailored advice on lifestyle improvement [34, 35]. A review of evidence from NHS Health Checks has demonstrated reductions in risk factors including BMI [36]. However, like the NHS DPP, uptake of the NHS Health Checks is lower among minority ethnic groups [19, 37].

The reduction of health inequalities in preventative programmes such as the NHS DPP and NHS Health Checks is amongst the key priorities of the NHS [20]. NICE guidance and service specifications for both programmes recommend developing culturally adapted interventions to increase uptake amongst populations likely to benefit most including those from minority ethnic groups [38–40]. Systematic review evidence of primary studies conducted in countries other than the UK, supports the effectiveness of culturally adapted prevention interventions for reducing/delaying the risk of developing T2D and CVD in ethnic minority groups [41–44]. However, the majority of the research exploring culturally adapted prevention interventions for reducing/delaying the development of T2D has been done in minority groups in the US, mainly African or Asian Americans [41, 45–47], Latin Americans [48], and Hispanic populations [43]. For CVD prevention, most adapted interventions in minority ethnic groups have been conducted in the US and China, and have focused on risk factors such as hypertension and smoking[44].

The growing burden of chronic diseases, specifically T2D and CVD, fuelled by the economic crisis and social inequalities [49], highlight the need for cultural adaptation of preventative interventions to target the specific barriers faced by ethnic minorities (33, 34). However, the extent of cultural tailoring of preventative interventions and evaluation in UK settings is unknown and has not been synthesised. As such, there is an evidence gap related to effective cultural adaptation strategies for developing T2D and CVD prevention interventions in minority ethnic groups [44].

The aim of this scoping review is to report the extent to which barriers, enablers and culturally adapted lifestyle interventions (diet or physical activity) intended to prevent or delay the onset of T2D have been explored in people from an ethnic minority background in the UK. In order to gain a greater insight into this area, it is also necessary to draw on culturally adapted lifestyle interventions implemented to prevent related chronic diseases such as CVD and Obesity which have the same predisposing lifestyle risk factors i.e., poor diet and physical inactivity. The objectives of this review are to: (1) report the barriers and enablers to intervention uptake, participation, and completion of diet and physical activity behaviour change interventions among minority ethic groups in the UK and link these to the COM-B behavioural change framework [50]; (2) describe the adapted interventions and link these to a cultural adaptation framework [51] and behaviour change techniques [52].

## Methods

This scoping review was conducted according to the Preferred Reporting Items for Systematic Reviews and Meta-Analysis Protocols Extension for Scoping Reviews (PRISMA-ScR) guidelines [53] and the methodological framework for conducting scoping studies developed by Arksey and O’Malley [54].

### Included Study Population

This review adopted the UK government’s definition for ‘ethnic minorities’ which uses the term to refer to all ethnic groups except the White British group [55]. Although, ethnic minorities also include white minorities, such as Gypsy, Roma and Irish Traveller groups, this review only focuses on populations at greatest risk of developing T2D, [50] namely African Caribbean, African and South Asian.

### Intervention Components

At the core, DPPs are behaviour change interventions targeted at dietary and physical activity behaviours [56, 57]. Therefore, lifestyle interventions with a diet and/or physical activity component with the aim of improving health outcomes, and which describe deliberate strategies used to enhance cultural relevance, were included. Diet components of interventions were defined as the manipulation of food or dietary intake directly (e.g., provision of food or nutritional supplement) or indirectly (e.g., nutrition education). Physical activity components of interventions were defined as the manipulation of physical activity directly (e.g., provision of exercise classes) or indirectly (e.g., education). The diet or physical activity components could be the sole focus of the intervention or delivered in conjunction with other components. As the national rollout of the NHD DPP in England was initiated in 2016, evidence on adaptations of the English DPP is anticipated to be low. Therefore, this review will include diet and physical activity interventions beyond those solely focused on diabetes prevention (e.g., those focused on CVD or Obesity prevention).

### Information Sources

The following databases were searched from January 2013 to January 2023: MEDLINE, Embase, PubMed Central and Cochrane Library. Since the NHS DPP was nationally introduced in 2016, the date range allowed for inclusion of preliminary studies. Published studies, of any design, were considered for inclusion. Commentaries and non-empirical papers were excluded. Reference lists of all included articles were hand searched to check that all relevant papers are included.

### Search Terms and Search Strategy

The search strategy was developed with a health librarian. Medical Subject Headings (MesH) terms from the National Library of Medicine were used to conduct the search and terms selected based on the population, intervention components and country. Search results were limited by language (English), Country (UK) and the last 10 years. 

### Study Selection

Initial title screening was conducted by TKC. Two reviewers then independently screened titles and abstracts (TKC and KK/JHP/SSO/CS/RC) followed by full text screening. Any uncertainties or disagreements about eligibility were resolved through discussion with a third reviewer. To be included, studies needed to meet all three inclusion criteria specified in A, one or both criteria specified in B, and none of the exclusion criteria:

### Inclusion Criteria

**A:** Population and intervention.

1. Adult population (aged ≥ 18 years) without diabetes
2. Strong representation of the following populations: African, African Caribbean and South Asian (Indian, Pakistani or Bangladeshi)
3. Lifestyle interventions (diet and physical activity, see intervention components)

**B**: Barriers and enablers or cultural adaptation.

1. Exploring cultural adaptation of lifestyle interventions defined as modifications that are responsive to the cultural needs of a local community and tailored to a cultural group’s traditional world views [58]. Interventions were considered culturally tailored if indicated in the text and/or if the cultural tailoring/adaptation aligned with the conceptual framework for tailoring prevention intervention[51].
2. Exploring barriers and enablers to intervention uptake, participation, and completion of lifestyle interventions (diet and physical activity, see intervention components).

### Exclusion Criteria

Review articles; meta-analyses; studies conducted outside of the UK; and articles in languages other than English.

### Data Charting Process

The following data were extracted by two independent reviewers (TKC and KK/JHP/SSO/CS/RC) and recorded on a standardised excel form.

- First author name and year of publication.
- Study location in the UK.
- Study aim and design.
- Study population (ethnicity, age, gender).
- Behaviour under study (e.g., physical activity or diet).
- Intervention or programme name.
- A brief description of the intervention (including length, frequency, and duration) reported theories of behaviour change used to underpin the intervention, mode of delivery (e.g., group based, online, face to face).
- A description of intervention outcome measures (if reported) and results (process and clinical outcomes).
- Barriers and enablers to uptake, participation and completion of interventions.

For studies exploring cultural adaptation:

j. Cultural adaptation strategies used (e.g., language).

### Collating, Summarizing and Reporting the Results

The focus of this review was on identifying and describing interventions or programmes that included cultural adaptations for the prevention of T2D and related conditions (CVD and Obesity) and barriers and enablers to engagement. Therefore, only a brief description of clinical outcomes (where reported) is provided.

### mapping Barriers and Enablers

Barriers and enablers identified from the included studies were mapped onto the COM-B (**C**apability + **O**pportunity + **M**otivation = **B**ehaviour change) theoretical model to understand and categorise key factors influencing engagement in minority ethnic groups [59]. The central principle of the COM-B model is that for any behaviour to occur there must be ‘capability (C)’ to do it; ‘opportunity (O)’ for it to occur; and enough ‘motivation (M)’ to perform it. Three reviewers (TKC, KK and RC) collectively mapped the barriers/enablers to the COM-B. Any disagreements/uncertainties were resolved by a fourth reviewer (FN).

### Mapping Cultural Adaptations to Behaviour Change and Adaptation Framework

To guide understanding of the theoretical underpinning of adaptations used to date, adaptations to interventions identified were linked to specific Behaviour Change Techniques (BCT’s) identified from a taxonomy of 93 Behaviour Change Techniques [50, 52, 59]. BCT’s are the active ingredient of an intervention (strategies) that can be used to change behaviour [52]. Examples include goal setting, action planning, feedback, prompts and cues. Two reviewers (KK and RC) independently mapped the intervention adaptations. Any disagreements/uncertainties were resolved in consultation with TKC and/or FN.

Adaptations linked to specific BCT’s were reported within the six dimensions of the conceptual framework for tailoring prevention interventions [51]: Cultural adaptations; Cognitive adaptation intervention; Affective-motivational adaptation; Environmental adaptation; Adaptations of program content and Adaptations of program form effectiveness.

### Synthesis of Results

Mapped barriers/enablers and cultural adaptations are presented in a narrative summary to summarise the characteristics of the included articles.

## Results

### Search Results

**Fig. 1:**
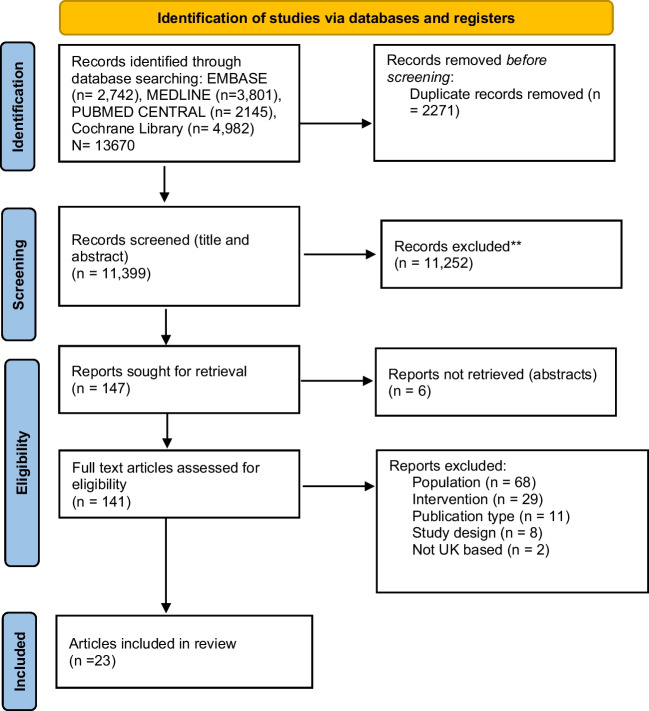
PRISMA flow diagram

A total of 13,670 records were identified, from which 2,271 duplicates were removed. Following initial title and abstract screening against the eligibility criteria, a total of 11,252 were removed before obtaining full text. One hundred and forty-one full text articles were retrieved of which 23 were eligible. The search process is presented in the PRISMA flow diagram in Fig. 1.

### Study design and Characteristics

Twenty-three studies in total were included in the review, 14 reported barriers and enablers only, four reported the design and delivery of culturally adapted interventions, and five reported barriers and enablers in relation to a specific adapted intervention (see Table 1).

**Table 1:** Characteristics of included studies exploring barriers and facilitators and/or describing culturally adapted interventions for minority ethnic populations in the UK

Author, Year	Location in the UK	Study Aim	Behaviour(s) under study	Study design	Population incl. ethnicity Age in years (mean ± SD/range Proportion Male/Female No. recruited	Other Stakeholders	Intervention name and description	Intervention length, duration & frequency	Mode of delivery/ Setting	Delivery personnel and training
Barry, 2021	Newham, East London	To understand general practice staff views and perspectives on the prediabetes diagnosis, supporting behaviour change, delivering lifestyle messages	Behaviour/lifestyle change leading to Diabetes Prevention	Qualitative—focus groups	Not patients Age: Not reported M/F: 10/15 N = 25	Primary Care Teams (incl. GP, nurses, Health Care Assistants, Pharmacists) serving an ethnically diverse population verse community. Ethnic makeup of the staff: African Caribbean—8, Asian-11, White-6	NA	NA	NA	NA
BhatnagarI, 2021	Manchester	To understand the barriers to and motivations for physical activity among second-generation British Indian women, with the ultimate aim of being able to provide recommendations to tailor physical activity interventions for second-generation British Indian women	Physical activity	Qualitative—semi-structured interviews	South Asian women 1st and 2nd generation British Indian women Age: 20–69 100% Female N = 28	NA	NA	NA	NA	NA
Brangan, 2018	Bristol	To explore in depth the experiences and perspectives of patients who received a telephone outreach call to invite them to take part in an NHS Health Check	Lifestyle improvement (including diet and physical activity	Qualitative—semi-structured interviews	GP registered patients identified as residing in areas of high deprivation or as potentially requiring cultural or language support. Ethnic makeup—16 White British and 8 were Black Caribbean, Black mixed, Bangladeshi, Somali, Jamaican and Polish Age: 40–66 M/F: 9/15 N = 24	NA	Telephone outreach intervention aimed to engage people from communities with potentially higher risk of cardiovascular disease, and/or less likely to take up a written invitation, with the NHS Health Checks programme—a programme aiming to prevent heart disease, stroke, type 2 diabetes and kidney disease, in adults aged 40–74 years using a combination of risk assessment communication of risk and risk management by tailored advice on lifestyle improvement	NA	Individual telephone calls	Trained community workers or interpreting service staff following receipt of motivational interviewing training
Cross-Bardell, 2015	East Midlands—Derby and Nottingham	To explore perspectives on enhancing physical activity and diet among South Asians in urban deprived communities at high risk of chronic disease and to inform development of culturally appropriate health promotion intervention	Diet and physical activity	Qualitative—one-to-one and family group interviews	South Asian (Pakistani, Indian, Bangladeshi/other South Asian) at high risk of diabetes and other chronic disease and families. Religion—Muslim, Sikh and Hindu Age: 41 (19–67) M/F: 11/23 N = 34	Bilingual South Asian dieticians, bilingual GPs in practices with majority South Asian populations, local authority exercise officers, community health promotion workers, a public health consultant, and local health service commissioners	NA	NA	NA	NA
Curry, 2015	Cardiff, Wales	to: (1) compare perceived PA and ST to objectively measured data; and (2) explore PA- and ST-specific contexts, experiences, and preferred sources of PA and ST amongst SA women in the UK	Physical activity	A mixed-methods study using interviews & accelerometery	South Asian (Pakistani & Bangladeshi) Age: 52.8 ± 10.1 100% Female N = 24		NA	NA	NA	NA
Derges, 2014	London	To examine the causal pathways that generated any intervention effects from the perspectives of local residents, who were involved as strategic partners in Well London’s design and delivery	Diet, Physical activity and mental wellbeing	Qualitative study (interview) nested within the Well London RCT	Londoners living in socioeconomically deprived neighbourhoods Population included African—18; Black British: 1; White British: 11; British Asian: 5; Indian: 4; Bangladeshi: 6; Pakistani: 1; European (Lithuania, Turskish): 4; Chinese: 3; Carribean: 6; Irish: 2 Age: 16–75 M/F: 16/45 N = 61 (intervention N = 45 (interviewed)		The Well London programme is a complex intervention comprising multiple components designed to improve the health of socioeconomically deprived Londoners using a community engagement model The interventions comprised a series of activities based around healthy eating, physical activity and mental well-being. This framework was used in each area but the delivery method varied according to local needs and priorities	1 year	Variable depending on activity	Co-ordinators and volunteers—variable dependent on activity
Eastwood, 2013	London (Central and North West)	The study aimed to explore the feasibility or acceptability of NHS Health Checks (the UK Department of Health’s nationwide initiative) in South Asian groups in London religious and community venues, as opposed to locally developed pilot screening programmes	lifestyle improvement—physical activity and diet	Qualitative—semi-structured interviews	Attendees (patients) (N = 12): recipients of the NHS health checks were invited to take part in the study. 3 were Bangladeshi British, and none were Indian British (the rest of the 8 unknown) Age: 30–67 (stakeholders) Age:27–49 (attendees) M/F: 3/9 (stakeholders) M/F: 6/6 (attendees) N = 12	religious and community leaders and clinical team, commissioners and GPs. Ethnic makeup: Bangladeshi British, Indian British and white British	The NHS Health Check programme A programme aiming to prevent heart disease, stroke, type 2 diabetes, and kidney disease, in adults aged 40–74 years using a combination of risk assessment communication of risk and risk management by tailored advice on lifestyle improvement	One-off 40-min single intervention	In person at religious or community setting (temple, mosque, Bangladeshi community centre)	Delivery by GP & organization at each venue by religious and community Leaders, volunteers, and champions
Emadian, 2017 (Diet)	London	To assess the dietary intake of a sample of South Asian men at risk of developing T2DM (as defined by their overweight/obesity status and ethnicity) and to understand the key factors that influence their dietary choices and eating behaviours	Diet	A mixed-methods cross sectional design, including assessment of dietary intake using UKDDQ, followed by semi-structured interviews	South Asian men (Indian, Pakistani and Bangladeshi ethnicity), with a body mass index of over 23.0 kg/m2 , not previously diagnosed with T2DM Age: 44.83 (9.90)/ Range: 25 – 64 100% Male N = 63 cross-sectional study N = 36 interviews		NA	NA	NA	NA
Emadian, 2017 (PA)	Greater London	To quantify physical activity (PA) and sedentary time (ST) using both self-report and objectively measured methods and explore factors influencing PA and ST using semi-structured interviews in a sample of overweight and obese South Asian men living in the UK	Physical activity	A mixed-methods cross-sectional design, including the quantification of PA using the IPAQ (ref) and accelerometery, followed by semi-structured interviews	South Asian men (Indian, Pakistani and Bangladeshi ethnicity), with a body mass index of over 23.0 kg/m2, not previously diagnosed with T2DM Age: 45.0 ± 9.79 100% Male N = 54 cross-sectional study N = 31 interviews		NA	NA	NA	NA
Garner-Purkis, 2020	London	To explore the experiences of participants and staff involved in an exercise referral scheme and to identify barriers and facilitators to the participation and the implementation of such projects	Physical Activity	Qualitative study—semi structured interviews	Physically inactive residents (i.e., completed 30 min or less of moderate activity a week) who were taking part or completed the in the 13 activity sessions of the Active Lifestyles for All (ALFA) project. Ethnic makeup: White 13 (52%); Mixed/multiple ethnic groups 2 (8%); Asian/Asian British 1 (4%); Black African 1 (4%); Black Caribbean 6 (24%); Other ethnic group 2 (8%) Age: 25–85 M/F: 1/24 Participants M/F: 3/7 Staff N = 35	Staff: referrers, mentors and other staff. Referrers worked for external organisations that referred individuals to the ALFA project and included GP’s and community workers	The Active Lifestyles for All (ALFA) project – a Community-based Exercise Referral Scheme (ERS) Aimed to increase physical activity the project involved trained health mentors working with physically inactive residents to develop individually tailored exercise and health plans for becoming active through a choice of a range of sport activities including yoga, fitness trampoline classes and Zumba	13 sessions delivered weekly for 12 weeks	In person with mentor with an option of group-based activities All activities took place at a Healthy Living Centre with exercise facilities, cafe, and space for social interaction in residential area off main road	Trained health mentors whose role was to support participants to engage in their selected sport activities
Horne, 2013	Not reported (UK)	To explore the barriers to initiating and maintaining regular physical activity (PA) among UK Indian, Pakistani and White British adults in their 60 s	Physical activity (initiation and adherence)	Qualitative—focus groups and interviews	South Asian (Indian, Pakistani, originating from the Indian subcontinent) (and White British (English, Welsh, with white British decent)) Age: 65.41/ Range: 60–70 European Age: 66.1/ Range 60–70 South Asian M/F: 15/58 European M/F: 16/29 South Asian N = 81 European N = 46 South Asian (N = 13 Indian, N = 33 Pakistani)		NA	NA	NA	NA
Horne, 2018	Northwest England	To determine the socio cognitive associations of intention to undertake physical activity among separate groups of Indian, Pakistani and Bangladeshi older adults aged > 60 using the Theory of Planned Behaviour	Physical activity	Cross sectional survey, informed by the Theory of Planned Behaviour	South Asian i.e. Indian, Pakistani and Bangladeshi older adults aged 60 years or older%) Age: 68.9 ± 6.48 M/F: 76/61 N = 138		NA	NA	NA	NA
Latif, 2016	London	To study the use and effectiveness of a low cost health promotion video intervention in impacting viewers’ knowledge and attitudes in regards to coronary artery disease (CAD)	Health Promotion—education	Before- and-after pilot study	Bangladeshi women aged 18 years or greater with Bengali as their first language Age: 53.7 (13.4)/ Range: 18–72 100% Female N = 18		An educational video on Coronary Artery Disease The video explaining basic heart physiology, coronary artery disease and its modifiable risk factors with practical guidance for prevention in line with the British Heart Foundation guidelines, but with a focus towards the Bangladeshi culture	One-off 8-min video	In person, group based at a Community centre—which holds regular women’s groups attended primarily by the local Bangladeshi women	A UK based consultant
Liljas, 2019	North London	To explore what influences ‘hard to reach’ older people practicing health promotion in later life; and to elicit the views of cross-sector professionals with experience of working with these sub-groups of older people, to help inform best practice on engagement of ‘hard to reach’ older people in health promotion	Healthy lifestyle	Qualitative interviews and focus groups	Community-dwelling older people from three ‘hard to reach’ groups including those aged ≥ 85 years, older people from BME groups, older people living in deprived areas Age: 65 to > 85 (NR for professionals) M/F: 8/11 (NR for professionals) N = 19 Participants (N = 4 Black African Caribbean, N = 4 Bangladeshi, N = 4 White European) N = 31 Stakeholders	Professionals with experience working with one or more of these sub-groups of older people including researchers/lecturers and directors), local authority practitioners, commissioners, managers and councillor; voluntary sector project officers, managers and trustee; and, NHS practitioners, social care manager	NA	NA	NA	NA
Morrison, 2013	Scotland (Glasgow and Edinburgh)	To explore the reasons for enrolling, experiences of participating and reasons for remaining in a family-based, cluster randomised controlled trial of a dietitian-delivered lifestyle modification intervention aiming to reduce obesity in South Asians at high risk of developing diabetes	Diabetes Prevention	Qualitative sub-study—interviews	South-Asians (± family member) at risk of T2D Age: NA M/F: 14/10 N = 24 (N = 20 participants, N = 4 Family volunteers)		NA	NA	NA	NA
Ochieng, 2012	North of England	To examine the barriers in accessing and utilising health promotion services	Healthy lifestyle	A questionnaire including included closed and open-ended questions	Newly arrived (< 5yrs) Black African migrant adults (> 18 years) literate in Arabic, English, French or Swahili Age: 18–65 M/F: 40/50 N = 90		NA	NA	NA	NA
Ochieng, 2013	West Yorkshire in the north of England	To describe African Caribbean' beliefs and perceptions about barriers to practising a healthy lifestyle	Healthy lifestyle- including diet and physical activity	Qualitative, interviews	African-Caribbean community Age Range: 22–60 M/F: 7/11 N = 18		NA	NA	NA	NA
Penn, 2014	Middlesbrough	To investigate Pakistani women’s perspectives of their behaviour, change and salient features of the culturally adapted NLNY programme	Diet, Physical activity and weight loss	A Qualitative sub-study of a feasibility evaluation of the adapted NLNY intervention—using group interviews	Pakistani women Age: 33.5/ Range: 29–45 100% Female N = 20		New Life New You (NLNY) The aimed to increased physical activity (PA), healthy eating and weight loss, in line with the UK NICE guidance. intervention consisted of Supervised physical activity sessions, reflection, behavioural counselling and advice centred on dietary information and action planning, and ongoing support	90 min – Weekly 8-week intervention, 12 months follow up	In person, group based (10–20) at Community venues in the areas of the town partly populated by the Pakistani community e.g. community halls, including school halls	The intervention was delivered by one Community Interest Company (CIC) founder member, who recruited other local Pakistani women to assist with intervention delivery. These women received training to qualify as fitness trainers or, in some cases, training to deliver the nutritional aspects of the intervention
Tomalin, 2019	Leeds and Bradford	Exploring places of worship as BAME public health settings (to deliver PH messages and services)	Healthy lifestyle- including diet and physical activity	Qualitative semi-structured interviews and focus groups	**Religious leaders’** representative from each main BAME faith tradition in the cities. Christian ministers (Anglican, Methodist and Pentecostal), an imam (the leader of worship in a mosque), Sikh members of Gurdwara committees, a Hindu temple chairman and the founder of a Buddhist centre Age: NA M/F: NA N = 19	Third sector organisation representatives working on wellbeing including two Muslim women organisations, an organisation working with asylum seekers, and an organisation focussing on mental health and Islam) Local authority public health professionals	NA	NA	NA	NA
Twohig, 2019	Sheffield	To explore the experience of diagnosis of pre-diabetes for people living in socioeconomically deprived areas, in order to gain insight into the interplay between people’s sociocultural environment, their understanding of, and response to, the diagnosis, and their decision to engage or not with the NHS DPP	Diabetes Prevention—diet, exercise, lifestyle and weight loss	Qualitative study, semi structured interviews	Aged > 18 years, had a coded diagnosis of pre-diabetes assigned within the preceding 12 months (by HbA1c test result of 42–47 mmol/mol), and had been offered referral to the NHS DPP. **Ethnic makeup**: sixty-nine per cent of participants were white British, 17% Asian, 9% African, and 5% Caribbean Age: 60/ Range: 37–81 M/F: 10/13 N = 23		NA	NA	NA	NA
Wallia, 2013 and Bhopal, 2014	Glasgow and Edinburgh, Scotland	To test the effectiveness of a family-based 3-year programme promoting weight loss and increased physical activity in individuals of south Asian descent living in the UK	Diabetes Prevention—diet, exercise, lifestyle and weight loss	Non-blinded, family-cluster randomised controlled trial	Indian or Pakistani aged 35 years or older at risk of type 2 diabetes and family Age: 52.8 (10.2) Intervention Age: 52.2 (10.3) Control M/F: 39/46 Intervention M/F: 39/47 Control N = 171 with IGT or IFG (N = 85 Intervention, N = 58 Control) N = 156 Family clusters (N = 78 Intervention, N = 78 Control)		Prevention of diabetes and obesity in South Asians (PODOSA) PODOSA is a family orientated, lifestyle intervention aiming to reduce weight and increase physical activity to, in the long-term, reduce the incidence of diabetes in people of Indian and Pakistani origin at high risk. The intervention involves consultations with a dietitian in which advise on achieving weight loss through a calorie-deficit diet and physical activity is provided and reinforced. Other components included Pedometers and annual group sessions, including a food shopping tour and brisk walking	15 visits from a dietician over 3 years	In person at home	Dietitians trained in venepuncture, anthropometric and blood pressure measurement, delivery of information, behaviour change using the stages of change model, and promotion of physical activity. Each family was mostly seen by the same dietitian throughout the study
Willis, 2016	Leicester	To assess the feasibility of delivering a faith centre-based pathway for screening and referral to group education sessions for T2D risk reduction	Diabetes risk reduction—education	A mixed methods feasibility study	South Asian background. Members of the public aged 35–75 without an existing diagnosis of diabetes Age: 56.2 (9.7) (Median, IQR) M/F: 99/103 N = 202		Screening & Walking Away from Diabetes Programme A structured education programme aimed at promoting walking in individuals identified with increased risk of developing T2D. The intervention involved screening using HbA1c assessments and a structured group education session for participants with an HbA1c value 6–6.4% (42–46 mmol/mol). The education session targeted knowledge and beliefs of diabetes risk, and promoted walking through increasing self-efficacy, discussing barriers and promoting self-monitoring and goal setting through pedometer use	One 3-h session	In person, group based Religious settings—one mosque, two Sikh Gurudwara and one Hindu temple	A GP; A Healthcare assistant—able to communicate in a variety of different local languages (Gujarati, Urdu, Punjabi)

Studies exploring barriers and enablers focused on diet and/or physical activity [60–75] or diabetes prevention [76–78]. Ten of the studies focused on South Asian populations [60–62, 64–66, 68, 70, 74, 77], seven reported a mixture of ethnicities [63, 67, 69, 71, 75, 76, 78] and two reported African & African Caribbean populations [72, 73]. Fourteen studies included mixed genders, with five reporting either men or women only participants [60–62, 66, 74].

For the studies reporting culturally adapted diet/physical activity interventions, six were conducted in South Asian populations [70, 74, 79–82], three in mixed populations including South Asian, African and African Caribbean [63, 67, 69]. Two studies included only female South Asian participants [74, 79], whilst the rest were mixed gender. Two of the studies evaluated adaptations of the NHS Health Check [67, 70], and others reported interventions aimed to prevent T2D [80–82], coronary artery disease [79], or improve diet/increase physical activity and reduce weight [63, 69, 74].

### Barriers and Enablers

Barriers and enablers influencing the uptake or implementation of diet and physical activity behaviour change, were mapped onto the COM-B model [50] (See Table 2). For both barriers and enablers, the COM-B components most frequently mapped were Social or Physical Opportunity (e.g., prioritisation of social and cultural commitments), Reflective Motivation (e.g., conflicts between religious beliefs and health practices) and Psychological Capability (e.g., understanding the intensity of physical activity needed to achieve health benefits). The following sections will provide a description of specific barriers and enablers that studies reported as influencing engagement in lifestyle changes for each COM-B component. The components are arranged in descending order, according to the number of studies that reported related factors.

**Table 2:** COM-B mapped barriers and enablers

COM-B component	Barriers	Article(s)	Enablers	Article(s)
Physical capability	Language • Language differences between health providers and their clients preventing sufficient access to informal and formal health promotion processes and services • Fluency in English, impeding/restricting participation and attendance in PA programmes	Ocheing, 2012	Communication in the participant’s chosen language(s)	Ocheing, 2012
	Comorbidities and poor health • Co-morbidities preventing physical activity; especially where there were coexisting, painful physical conditions limiting mobility • Co-morbidities posed challenges and issues with unsuitable referrals resulting in classes with an extreme mix of abilities	Horne, 2013 Liljas, 2019 Twohig, 2019 Garner Purkis, 2020		
	Experience of undertaking exercise • Lack of experience into how to exercise at an intensity that is moderate or vigorous	Curry, 2014		
COM-B component	Barrier	Article(s)	Enablers	**Article(s)**
Psychological Capability	Lack of education e.g., CVD awareness • Older generations in Sikh and Hindu settings less likely to exercise than younger (and ‘better educated’) members • Poor health literacy	Eastwood, 2013 Tomalin, 2019, Barry, 2021	Disease awareness—through knowledge and personal experience • A good understanding of T2DM and the role of PA in reducing its effects • A good understanding of the main lifestyle-related risk factors contributing to the development of T2DM • An understanding of the increased diabetes risk associated with Asian ethnicity • Understanding of term 'pre-diabetes' • Understanding and fear of T2DM consequences (including diabetes, medication, and diabetes complications) motivating patients to change their lifestyles	Eastwood, 2013 Emadian, 2017 (Diet) Emadian, 2017 (PA)* Morrisons, 2013 Batanghari, 2021 Curry, 2014 Penn, 2014 Barry, 2021 Twohig, 2019
	Understanding of physical activity • Conceptual understanding and contextualisation of PA not coinciding with those of researchers/health professionals/policy makers e.g., participants conceptualising PA as ‘keeping busy’ or ‘moving around’ • Lack of familiarity with the UK guidelines for PA leading to poor perceptions on types of physical activities that meet recommendations e.g., housework • Over- or under-estimated their physical activity level Lack of knowledge how to exercise at an intensity that is moderate or vigorous	Curry, 2014 Emadian, 2017 (PA)	Understanding of physical activity Clearly defining and emphasising the intensity of PA needed to achieve health benefits including the provision of real-life examples of moderate and vigorous intensity activities in which women can engage	Curry, 2014
	Dietary advice not culturally appropriate • Lack of nutrition labels on some sweet foods, specifically intended for Asian festivals • Advice not accommodating their more diverse diets international food diet—dietary advise based primarily focussed on Western diet • Existing healthy lifestyle principles and strategies perceived not to have taken the identity, values and beliefs of African Caribbean people	Morrisons, 2013 Ocheing, 2012 Ocheing, 2013 Liljas, 2019 Penn, 2014	Inclusive health promotional advice • Provision of appropriate education while taking into considerations individual’s values and beliefs e.g., traditional African Caribbean diet • Positive images and information supportive of African Caribbean community beliefs and values	Ocheing, 2013
	• Perception that cooking healthy vegetarian food was a challenge (with regards to the Gujarati population)	Eastwood, 2013	Confidence to make changes	Emadian, 2017 (Diet)
	Awareness • Unaware of the intervention	Garner Purkis, 2020		
	Training (Delivery) • Lack of staff skills in dealing with medical conditions that required special care	Garner Purkis, 2020		
COM-B component	Barriers	Article(s)	Enablers	**Article(s)**
Physical Opportunity	Location • Inconvenient timing of the sessions • Access issues with regards to the location of the project centre with its residential positioning	Garner Purkis, 2020	Location • Using local facilities • Places of Worship embedded within communities, providing a safe and trusted space • Using local and informal settings (e.g. homes) that provide cultural access and engagement reducing barriers • Arranging attendance prompts as part of the women’s usual routine e.g. community venue adjacent school	Tomalin, 2019 Cross-Bardell, 2015 Penn, 2014 Batanghari, 2021 Curry, 2014
	Socio-economic disadvantage and financial constraints • Food choices constrained by poverty and financial stresses • Affordable food often poor-quality i.e. high in sugar/ salt • Cost of gym membership • The cost of attending sessions (once the free and subsidised period had expired) • Poverty and deprivation	Ocheing, 2013 Tomalin, 2019 Barry, 2021 Cross-Bardell, 2015 Twohig, 2019	Cost • Providing free or subsidised exercise sessions incentivised the participation in the project • Providing free access to facilities • access to affordable and nutritious food,	Garner Purkis, 2020 Derges, 2014 Liljas, 2019
	Transport • Difficulty affording public transport was a frequently cited barrier to attending intervention e.g., the NHS DPP	Twohig, 2019 Garner Purkis, 2020	Transport • Family able to provide transport	Liljas, 2019 Twohig, 2019
	Poor housing • Food choices constrained by poor quality housing—impacting on preparing meals and exercising at home	Barry, 2021	Environmental factors • Socially cohesive environment: get to know neighbours, safe and well maintained	Derges, 2014
	Obesogenic environment • Food choices constrained by area deprivation • Social deprivation, the easy availability of cheap fast food perpetuating obesity • the high number of fast-food outlets in the area reflects the demand for quick, cheap food and its cultural acceptance	Barry, 2021	Tailoring activities • Providing a variety of classes that is suitable for participants’ differing needs and health conditions motivated the continuation in physical activity • Considering walking as the most feasible and culturally appropriate physical activity (South Asian) that had the potential to include a social element and involve friends and family • Using aids or seated exercise to minimise fear of falling	Garner Purkis, 2020 Cross-Bardell, 2015 Horne, 2013
	No Physical education (PE) lessons in school	Batanghari, 2021	Availability of interventions • Opportunity afforded by availability of intervention programme	Penn, 2014
	Time constraints due to family, work and domestic commitments • Long working hours and physically demanding employment leaving little available time • Organizing childcare to allow evening exercise • Caring responsibilities for children or older or sick relatives	Curry, 2014 Eastwood, 2013 Emadian, 2017 (Diet) Emadian, 2017 (physical activity) Morrisons, 2013 Cross-Bardell, 2015 Garner Purkis, 2020 Twohig, 2019	Tailored advice • Providing relevant and tailored advise based on personal experiences, if possible, rather than limited to facts about health benefits of physical activity and healthy diet	Penn, 2014
	Time constraints due to social and cultural commitments • Busy social lives due to cultural activities e.g., attending family events, ceremonies, etc. where it is hard to get out of them • High frequency and lengthy duration of cultural events including religious events and weddings which play an integral role in South Asian communities	Eastwood, 2013 Emadian, 2017 (Diet) Morrisons, 2013 Penn, 2014	Recruitment • Phone calls made booking appointment easy and immediate. Calls also meant could asked questions – though some prefer to be sent letters to be able to take time to read about opportunity	Brangan, 2018
	Religious beliefs • Religious practices of Muslim participants, such as fasting, could become an added barrier to activities • Islam influences – non mixing with men	Horne, 2013 Morrisons, 2013 Batanghari, 2021	• Preference for face-to-face contact	Brangan, 2018 Cross-Bardell, 2015
	Poor weather in the UK • Limiting outdoor activity • Scotland’s challenging climate and a related reluctance to outdoor exercise (e.g., walking)	Emadian, 2017 (physical activity) Morrison, 2013 Penn, 2014	Language • Translated information into the languages spoken by the majority of the ‘newly arrived’ Black African migrant families, including Arabic, French and Swahili Consideration of language differences	Morrison, 2013 Ocheing, 2012 Cross-Bardell, 2015 Liljas, 2019
	Language • Dissemination of health promotion information to Black African migrant communities only in the English language	Ocheing, 2012	Deliverers • Continuity in dietitians positive and trusting relationships • Professional support	Morrison, 2013 Horne, 2013
	Setting related barriers – places of worship • Places of worship not always having appropriate space or facilities	Tomalin, 2019	Intervention components – follow-up • The sense of care and continued follow-up was an essential factor to participant engagement	Garner Purkis, 2020
COM-B component	Barrier	Article(s)	Enablers	**Article(s)**
Social Opportunity	Social and cultural commitments • Prioritisation of social and cultural commitments leading over lifestyle changes e.g., religious events, family events/weddings, ceremonies, etc. where it is hard to get out of them • High frequency and lengthy duration of cultural events which play an integral role in South Asian communities but are major contributors to the overconsumption of less healthy food • Social responsibilities e.g., providing hospitality were reported as important barriers to persevering with agreed dietary goals	Eastwood, 2013 Emadian, 2017 (Diet) Morrisons, 2013 Penn, 2014	Creating a safe and comfortable space • Offering the space to exercise in a safe and comfortable environment due to it attracting people of all shapes and abilities • The participation of likeminded companions in group physical activity facilitated the enjoyment and integration to physical activity scheme	Garner Purkis, 2020
	South Asian Diet • High volume of ghee (clarified butter), oil and fried foods in traditional South Asian cooking • South Asian heritage diets leading to consumption of high levels of sugar and fat	Eastwood, 2013 Emadian, 2017 (Diet) Tomalin, 2019	Cultural and religious considerations • Availability of group sessions for specific genders e.g. the provision of a women-only facilities • Culturally sensitive at leisure facilities	Horne, 2013 Liljas, 2019 Penn, 2014
	Cultural norms about food and eating • The role of food in community functions and faith is a challenge to adherence to healthy diet • Views or practices of family members restricting food choices e.g., older generations preferring traditional food cooked in the conventional manner rather than lower fat recipes • The importance of specific foods within South Asian cultures e.g., home-made sweets and traditional food preparation techniques for which abstinence maybe viewed unfavourably • Communal eating of traditional ‘Asian’ food central to their social and cultural lives, and key to their standing, particularly concerning hospitality with others thus presenting difficulties of making and maintaining dietary changes	Emadian, 2017 (Diet) Eastwood, 2013 Barry, 2021 Cross-Bardell, 2015 Twohig, 2019	Cultural acceptance • Understanding and acceptance of social roles, social and cultural issues that were pertinent in Pakistani women as providers also from Muslim community • Making reference to Islamic culture during recruitment, sessions, and engagement strategies resulting to the women comfortable and safe • Recognition of participants double or triple identities in healthy living advice which may be formed from African, Caribbean and British backgrounds	Penn, 2014 Ocheing, 2013
	Culturally inappropriate (professional) dietary advice • Dietary advice not considering cultural differences • Perception that healthy eating meant giving up part of their ethnic identity	Morrisons, 2013 Ocheing, 2013 Liljas, 2019 Tomalin, 2019 Cross-Bardell, 2015	Religious beliefs • Adherence to religious beliefs promoting healthy lifestyle, e.g., forbidding alcohol consumption, smoking or immoderate food consumption	Eastwood, 2013 Batanghari, 2021
	Social exclusion • Having different priorities from the societal understanding of what constitutes a healthy lifestyle e.g., non-inclusion of African Caribbean foods as part of a healthy diet, and promoting only White British values and beliefs about healthy lifestyle • lack of consideration of religious and cultural factors which often influences people’s interpretations of health issues	Ocheing, 2013	External sources of encouragement • **T**rusted and influential religious or community leaders who are well informed about the health issues facing their communities and are already involved in some health-related work • A link worker from their local community working alongside a health promotion specialist as a strategy to highlight some of their challenges in accessing health promotion advice • Relevant external organisations	Eastwood, 2013 Tomalin, 2019 Batanghari, 2021 Derges, 2014
	Gender roles and expectations • Domestic pressures and expectation for women to prioritise family and domestic duties (e.g., family care and cooking) leading to lack of opportunity to participate in physical activity/sports • Lifestyle change was seen as particularly difficult for women, the demands of family life and work were prioritised above self-care • Social restriction for women in some communities e.g., Bangladeshi • The tendency for women in the family to be the main cooks, affecting the ability for men to make changes to diet and leading to a perceived lack of control over their food shopping and preparation • South Asian men are more dependent on their families compared to other men in the UK	Barry, 2021 Batanghari, 2021 Horne, 2013 Twohig, 2019 Emadian, 2017 (Diet) Emadian, 2017 (PA) Eastwood, 2013	Health care professionals and delivery personnel • Friendliness and lack of duress when extending invitations • Social support from mentors (delivering the intervention) creating a sense of responsibility and loyalty to continue with the project • Peers or trusted General Practitioners • 'Enthusiasm' of healthcare provider where diagnosis (of prediabetes) is presented as a health priority and urging their patient to attend the NHS DPP	Brangan, 2018 Garner Purkis, 2020 Twohig, 2019 Liljas, 2019
	Religious beliefs and commitments • Religious festivals and practices also affected their ability to maintain regular attendance and participation in PA at key times in the calendar • Faith-based responsibilities were reported as important barriers to persevering with agreed dietary goals	Horne, 2013 Morrisons, 2013	Environmental factors • Socially cohesive environment: get to know neighbours, safe and well maintained	Derges, 2014
	Religious beliefs • Settings related barriers—The impact of moral and social dynamics in using places of worship e.g., women are less likely to attend the mosque than men as they are not obliged to pray there, and often there are no separate facilities for them • Islam influences – prescribing modesty	Tomalin, 2019 Batanghari, 2021	Intervention components with social elements • Considering **walking** as the most feasible and culturally appropriate physical activity that has the potential to include a social element and involve friends and family • Addressing social aspects to successfully engage BME older people in health promotion	Cross-Bardell, 2015 Liljas, 2019
	Referral processes • Low engagement of stakeholders e.g., poor engagement of GPs in the referral process • ‘Scepticism’ of healthcare professional delivering diagnosis: sceptics minimised or dismissed the diagnosis as irrelevant leading to patients declining referral • Referral type: External source of referral i.e., GP rather than self-referral	Garner Purkis, 2020 Twohig, 2019	Positive health promotion messages in both the media and while at school influencing change amongst South Asians who are born and raised in the UK	Batanghari, 2021 Emadian, 2017 (physical activity)
	Cross-Cultural comparisons • Prioritisation of academic achievement over sports engagement within South Asian families during childhood and adolescence • Higher importance placed on physical activity in the UK compared to less importance being placed on PA in South Asian countries • Differences to exposure to health promotional messages (older generations)—Asian television channels having no physical activity message, with the exception of yoga	Batanghari, 2021 Emadian, 2017 (physical activity)		
	Family influence • Needing family members across generations to engage in modifying dietary behaviours • Views or practices of family members restricting food choices e.g., older generations preferring traditional food cooked in the conventional manner rather than lower fat recipe • Negative influence from family and peers e.g., dropping out of health promotion classes	Emadian, 2017 (physical activity) Cross-Bardell, 2015 Eastwood, 2013 Liljas, 2019	Family and peer support • Activities that fitted around family life • Family support with respect to food choice and preparation with wives • Motivational effects of sharing attempts at health promoting activity with family and friends • Exercising in a group • Involvement of community peers in facilitation and motivation • Social support in the form of participants’ relationship with their mentors and fellow participants • Peer encouragement including becoming ‘champions’ in promoting the intervention • Purposeful social grouping by presenting an opportunity for support and social networking	Emadian, 2017 (Diet) Liljas, 2019 Cross-Bardell, 2015 Emadian, 2017 (physical activity) Horne, 2013 Garner Purkis, 2020 Penn, 2014
	Language—of fluency in English, which impeded/restricted participation and attendance in PA programmes - Language differences between health providers and their clients preventing sufficient access to informal and formal health promotion processes and services, with the result that the real diversity of health promotion needs in the communities remained largely hidden - Support networks not being available to non-English speakers	Horne, 2013 Ocheing, 2012 Liljas, 2019	Language • Collaborate of health service practitioners with local migrant Black African communities to develop good and effective translating and interpreting services that are embedded within the communities	Morrison, 2013 Ocheing, 2012 Cross-Bardell, 2015 Liljas, 2019
	Racism, prejudice and discrimination - Experiences of racism, prejudice and discrimination – Racism was considered to have a direct effect on socio-economic position, health status and overall well-being - Oppression Racist attitudes from the community	Ocheing, 2013 Tomalin, 2019	Overcoming mistrust by taking a more person centrered approach • Overcoming mistrust from BME participants by spending time for patients to communicate their narrative, free from judgement, and feel that they have been heard • Mutual trust between the healthcare provider and patient, with knowledge of their social circumstances and cultural context • Encouraging the patient to decide what was possible, alongside showing an interest and offering kindness, was reported as key in this approach	Liljas, 2019 Barry, 2021 Penn, 2014
	Socio-economic disadvantage - Lack of social opportunities, experiences of deprivation and socio-economic disadvantages especially for African Caribbean men leading to limited time and personal capability to even begin to consider preventive healthcare measures owing to the constant pressure of their adverse social circumstances e.g., unemployment	Ocheing, 2013 Tomalin, 2019 Twohig, 2019	Social and economic considerations • Considering healthy lifestyle as a component of a number of public or social service facilities including having access to employment, relevant education, health care and good housing, with equality and fairness in their delivery	Ocheing, 2013
	Normalisation of diabetes within communities meant the impact of being diagnosed with pre-diabetes was lessened	Twohig, 2019	Props • positive views on the use of pedometers for motivation	Cross-Bardell, 2015
COM-B component	Barriers	Article(s)	Enablers	**Article(s)**
Reflective Motivation	Confidence Lack of confidence e.g., embarrassment about going to the gym	Penn, 2014	Confidence • Building confidence and autonomy which allows involvement in decision-making to improve confidence and self-esteem	Derges, 2014
	Lack of motivation to continue dietary changes	Emadian, 2017 (Diet)	Intrinsic motivation • Self-referred participants appeared to be more motivated and engaged • Personal responsibility of health	Emadian, 2017 (Diet) Emadian, 2017 (physical activity) Horne, 2013
	People with comorbidities are less inclined to take up regular PA due to fear of exacerbating pre-existing health conditions and causing harm or feeling they do not have much control over their ability to participate in PA	Horne, 2013	Acknowledging the importance of taking time out for oneself	Batanghari, 2021
	Concerns over negative perception of appearance while exercising	Batanghari, 2021		
	The perception that being healthy is time-consuming	Eastwood, 2013	Perceived benefits of healthy lifestyle • Weight loss, improving bone/joint function • Maintenance of good health and independence as they age	Curry, 2014 Horne, 2013 Morrison, 2013 Liljas, 2019
	• Perceived concern that for women, exercising outside alone is unsafe • Concerns about personal safety, while travelling to the programme through areas seen as unsafe	Batanghari, 2021 Curry, 2014 Twohig, 2019	Diagnosis of pre-diabetes • which can act as a brief intervention to promote positive lifestyle change	Twohig, 2019
	Asymptomatic ‘pre-condition’ (pre-diabetes) insufficient motivator to make changes	Twohig, 2019		
	Religious beliefs about disease Religious belief that views illness as ‘God’s will’, a punishment as the result of sin or karma, rather than believing a health intervention can change outcome	Tomalin, 2019	Disease awareness and hope of receiving information to guide their lifestyle changes • Awareness and experiences of family or friends with illness or complications of diabetes • known family history of diabetes and the desire to better understand ways to mitigate risks	Twohig, 2019 Morrisons, 2013 Batanghari, 2021
	Personal circumstances and socio-economic disadvantage • At an individual level, personal circumstances may prioritise over healthy intervention • Apathy and indifference to health advise associated with socio-economic circumstances amongst BME groups living in deprived areas who believed little could be done about their health • Limited time and personal capability to even begin to consider preventive healthcare measures owing to the constant pressure of their adverse social circumstances e.g., unemployment	Garner Purkis, 2020 Liljas, 2019 Ocheing, 2013 Tomalin, 2019 Twohig, 2019	Social and economic considerations • Considering healthy lifestyle as a component of a number of public or social service facilities including having access to employment, relevant education, health care and good housing, with equality and fairness in their delivery • Not making healthy lifestyles about ‘social order’ and ‘control mechanisms’ to promote certain behavioural attributes, but instead designed them to encourage community empowerment, harmony and understanding within and with other ethnic groups	Ocheing, 2013
	Racism, prejudice and discrimination • Oppression and racist attitudes from the community considered to have a direct effect on socio-economic position, health status and overall well-being	Ocheing, 2013 Tomalin, 2019	Wellbeing and social cohesion • Wellbeing seen as catalyst that enables participation through sense of personal agency • Improvements in health practices enhanced sense of wellbeing and associated social cohesion	Derges, 2014
	Religious and cultural beliefs and practices • Prescribing modesty and gender restrictions • Religious practices of Muslim participants, such as fasting, a possible barrier to activities • perceiving prayer time (typically three to five times per day) as adequate duration and intensity of physical activity	Batanghari, 2021 Horne, 2013 Curry, 2014	Intervention delivery • Intervention with an emphasis on spoken communication, multilingual spoken content (e.g., using personal stories) and delivery, including use of personal experiences • Time for the patient to communicate their narrative, free from judgement, and feel they have been heard	Barry, 2021 Cross-Bardell, 2015
	Activities which are out of touch with local needs – ‘fun’ activities less successful than those with direct relevance e.g., stress management (context)	Derges, 2014	Intervention delivery • Focus on small behaviour changes occurring incrementally over time	Barry, 2021 Penn, 2014
	• Exercise: Prioritising traditionally cooked meals from scratch each day, which takes approx. 2 to 3 h	Curry, 2014	Intervention delivery • *Continuity* in dietitians—positive and trusting relationships • The sense of care and continued follow-up was an essential factor to participant engagement • Participants relationship with their mentors created a sense of responsibility and loyalty to continue with the project	Brangan, 2018 Morrison, 2013 Garner Purkis, 2020
	Personal belief that works and family commitments should be priority	Emadian, 2017 (Diet) Emadian, 2017 Batanghari, 2021	Intervention delivery • Scare tactics (depicting dire outcomes if no action was taken)—this approach *had to be tailored* and timed correctly for each patient as these tactics had negative consequences, such as stigmatising people, which may prevent them from returning for review, or engaging in behaviour change or lifestyle interventions	Barry, 2021
			Peers/social support • Motivational effects of sharing attempts at health promoting activity with family, friends and community peers • Purposeful social grouping, and the opportunity this presented for women to talk together and support each promote greater confidence within participants’ perceived role and decrease isolation and depression	Emadian, 2017 (physical activity) Horne, 2013 Cross-Bardell, 2015 Garner Purkis, 2020 Liljas, 2019 Penn, 2014
COM-B Component	Barriers	Article(s)	Enablers	**Article(s)**
Automatic Motivation	Cultural norms about food and eating Difficult to change old eating habits	Emadian, 2017 (Diet)	Individual parameters such as **self-control**	Eastwood, 2013
	Social isolation • Unwillingness to socialise (based on drive)	Liljas, 2019	Fear of consequence**s** (including diabetes, medication, and diabetes complications) motivating patients to change their lifestyles	Barry, 2021 Penn, 2014 Twohig, 2019
	Social exclusion • Promoting Western diet as healthier to African Caribbean (Anger, feeling of disrespected)	Ocheing, 2013	Cultural acceptance • Cultural acceptance through references to Islamic culture during recruitment, sessions, and engagement strategies resulted to participants feeling comfortable, safe, happier, less isolated, and not depressed	Penn, 2014
	• The mainly English-speaking staff was a factor that added to their anxiety and discomfort in using health promotion services	Ocheing, 2012	Sense of achievement and beneficial effects	Penn, 2014

PA = Physical Activity

### Social Opportunity [60–78]

Amongst South Asian communities, the high frequency and lengthy duration of social events (e.g., weddings, ceremonies etc.) associated with consumption of food high in fat and sugar, was cited as a barrier to making lifestyle changes. These events, integral to South Asian culture, were prioritised over other activities such as exercise. Social norms in relation to gender roles and expectations were also cited as a barrier. For women, the expectation to prioritise family and domestic responsibilities, limited time available to engage in structured physical activity. Men felt they could not make healthy dietary decisions as it was the role of the women to shop for groceries and prepare food.

Religious and cultural norms amongst South Asian, African and African Caribbean communities (alongside their British background) were also cited as important determinants. For example, adherence to some religious beliefs (e.g., forbidding alcohol consumption and moderate food consumption) was cited to promote a healthy lifestyle, whilst other religious practices (e.g., fasting) were seen as potential barriers to maintaining regular attendance and participation in physical activity. The prescription of modesty in some cultures and religious beliefs also meant that women were less likely to participate in lifestyle interventions with mixed genders. Availability of intervention sessions for specific genders, e.g., women-only exercise facilities, was viewed as an important enabler.

Socio-economic challenges were cited as barriers for engaging in lifestyle interventions, particularly amongst African and African Caribbean participants. Experiences of racism, prejudice and discrimination were viewed to have a direct effect on socio-economic positioning, health status and well-being. Such experiences were seen to have a direct effect on participants willingness to engage in health promotion activities. In addition, the pressure of adverse social circumstances, e.g., unemployment, affected capacity to engage in behaviour changes. As such, consideration of socio-economic circumstances including access to employment, relevant education, health care and good housing, with equality and fairness was seen as a catalyst to enabling participation in lifestyle interventions.

### Physical Opportunity [60–63, 65–78]

Location and session timing were important determinants, where delivery of interventions in convenient and trusted local facilities (e.g., places of worship) and ease of access as part of usual routines (e.g., physical activity/exercise venues adjacent to schools) facilitated engagement. The use of local and informal settings such as homes and places of worship, were important for facilitating a safe, culturally acceptable and trusted space.

Engagement in positive dietary and physical activity changes was constrained by financial insecurity and poverty stresses. The cost of public transport was frequently cited as a barrier to accessing interventions. Environmental barriers included poor weather limiting outdoor activities, high number of fast-food outlets and availability of cheap (highly processed) food near peoples’ homes. The provision of free or subsidised exercise facilities/sessions, access to affordable and nutritious food and safe and well-maintained environments were seen to incentivise participation.

### Reflective Motivation [60–63, 65–71, 73–78]

Barriers and enablers mapped to this category, were shaped by reflections on self-perceptions, and social, cultural and religious experiences and beliefs (reported under Social Opportunity). Cited barriers included lack of confidence (e.g., a negative perception of appearance while exercising), fear of exacerbating pre-existing health conditions, and for women, concerns of personal safety whilst exercising outside the home or travelling to intervention venues. Purposeful social grouping was therefore important. The motivational effects of sharing attempts at health promoting activities with family, friends and community peers were identified as important for both engagement and promoting greater confidence amongst participants.

Religious views about disease (e.g., diabetes) were sometimes stated as a barrier. For example, an external locus of control in which illness was perceived as ‘God’s will’ or a punishment as the result of sin or karma, and for which a health intervention would not change the outcome. In South Asian communities, the higher prioritisation of academic achievement over sports engagement during childhood and adolescence, was cited as a barrier. These are important considerations when developing interventions for second-generationversus first generation participants. In addition, except for yoga, there is limited exposure to health promotional messages for physical activity for older generations who primarily watch Asian television channels.

### Psychological Capability [60–63, 66, 70–78]

The lack of knowledge about UK physical activity guidelines and how to exercise at required intensities, was stated as a barrier which led to a mismatch between participants and health professionals conceptual understanding and contextualisation of physical activity. Thus, clearly defining the intensity of physical activity needed to achieve health benefits, including real-life examples was identified as a key enabler.

Education and health literacy (e.g., a good understanding of T2D, its complications, risk factors and the role of diet and physical activity in reducing its effects) was identified as an important enabler for engaging in heathy behaviour changes especially amongst older generations in Sikh and Hindu communities.

A lack of culturally appropriate dietary advice was a barrier to knowing how to make appropriate dietary changes. Traditional food plays a central role in South Asian, African and African Caribbean communities. It is considered by some to be part of their ethnic identity. The exclusion of their traditional foods in conceptualisations of what constitutes a healthy diet was perceived by some as a form of social exclusion, where only White British values and beliefs about healthy lifestyle are promoted. Thus, the provision of appropriate education and advice that reflected participants identity, values and beliefs and displaying positive images and information supportive of such beliefs and values were important for encouraging change.

### Automatic Motivation [66, 70–74, 76, 78]

Fear of consequences including diabetes, medication, and diabetes complications were motivators to lifestyle changes. Socio-economic disadvantages and challenges were associated with feelings of anger and disrespect and negatively affected the willingness to participate in lifestyle interventions. In addition, for migrant African ethnic minorities using health promotion services, the presence of mainly English-speaking health care providers and the lack of translators added to anxiety and discomfort. References to other cultures and beliefs during recruitment, sessions, and engagement strategies were seen to promote cultural acceptance leading to participants feeling comfortable, safe, happier, and less isolated.

### Physical Capability [61, 63, 65, 71, 72, 78]

The lack of intervention resources in different languages including health providers and health information, limited attendance in lifestyle interventions especially for African migrants. The lack of experience in how to exercise at an intensity that is moderate or vigorous was cited as a barrier, as was co-morbidities which hindered undertaking physical activities.

### Linking Cultural Adaptations to Behaviour Change Techniques

The following sections provide a description of intervention adaptations which have been linked to strategies known to influence behaviour change (BCT’s) [52]. The adaptations and their strategies have been grouped and reported within dimensions of a framework of cultural adaptations [51] (See Table 3).

**Table 3:** Tailored intervention components and related behaviour change techniques

	Brangan, 2018	Derges, 2014	Eastwood, 2013	Garner-Purkis, 2020	Latif, 2016	Penn, 2014	Wallia 2013 & Bhopal, 2014	Willis, 2016
Environmental adaptation: Ecological aspects of the community, for example implementing in homes instead of at a clinic	Recruitment approach Telephoning patients (instead of sending letters) amongst communities known to be at higher risk of cardiovascular disease, and/or less likely to take up a written invitation, to engage them with the NHS Health Checks programme BCTs* Prompts/cues		Intervention setting Religious or community setting—two Hindu temples (attended by a Gujarati Indian population) and a mosque (attended mostly by a Bangladeshi Muslim population) and a Bangladeshi community centre BCTs Restructuring the physical environment Restructuring the social environment	Intervention setting Non- gym setting for intervention delivery Recruitment approach Multi-method of recruitment including Self-referral, referral from the NHS including GPs, pharmacies and mental health services, public health, and community organisations BCTs Social Support unspecified Credible source, Restructuring the physical environment Restructuring the social environment	Intervention setting A community centre which holds regular women’s groups attended primarily by the local Bangladeshi women of the surrounding council estates BCTs: Restructuring the physical environment Restructuring the social environment	Intervention setting Local, convenient, and familiar venue (community venues in the areas of the town partly populated by the Pakistani community) Initial access to a community gym, exclusive for the intervention (later stopped) BCTs: Restructuring the physical environment Restructuring the social environment	Intervention setting Home setting to encourage family involvement especially those who prepare the family meal. Home visits were flexible, including on weekends, and avoiding Muslim or Hindu fasting periods. Participants were also seen at their preference, in NHS premises, and in voluntary organizations and workplaces Recruitment approach (1) NHS settings (2) Local south Asian organisations and individuals—talks delivered in places of worship, community organizations in Hindi, Urdu, Punjabi and English (3) Participants encouraged to refer friends and family BCTs Social support Credible Source Restructuring the physical environment Restructuring the social environment Identify associated with changed behaviour	Intervention setting Faith Centres—one mosque, two Sikh Gurudwara and one Hindu temple BCTs Restructuring the physical environment Restructuring the social environment
Affective-motivational adaptation: Characteristics related to gender, ethnic background, values, traditions, religious background, and socioeconomic status AND Cultural adaptation—Tailoring the intervention to meet community’s worldview and lifestyle	Ethnic background Where possible matching outreach caller main language with that of the patient called BCTs Credible source Restructuring the social environment	Values and traditions Use of co-ordinators and volunteers dependent on activity ‘Intervention delivery method varied according to local needs and priorities’ BCTs Social Support practical Restructuring the social environment	Values and traditions In one setting community volunteers and ‘champions’, i.e., organizers or leaders helped administer the programme and inspire community participation BCTs Credible Source Restructuring the social environment	Socioeconomic status Sport activities sessions provided free of charge for 13 weeks. Subsequently, sessions were offered at a reduced rate BCTs Material incentive (behaviour)	Ethnic background An educational video on coronary artery disease in Bengali language BCTs Information about health consequences	Ethnic background, values, traditions, religion Consistency of the same provider and who was from a similar ethnic background and could appreciate cultural and religious context Gender and values Women only intervention. Community based recruitment through women-only assessment events organised in consultation with community health champions and Snowballing recruitment by participants themselves Socioeconomic status Sessions provided at no cost to the participants for up to 6 months and from then on at low cost BCTs Social support unspecified Practical Social Support Credible Source Restructuring the social environment Material incentive (behaviour)	Ethnic background Registered dietitians who were aware of the relevant food and physical activity practices of South Asians and who collectively could speak English, Punjabi, Urdu and Hindi and knew about Sikhism, Islam and Hinduism Values same dietitian throughout the study to encourage rapport with one professional Family cluster—consisting of the participant plus any family volunteers. First degree relatives (parents, siblings, children) living in the same city. Cooperation of the household’s main cook was required Religious background Deletion of foods taboo to Muslims like pork and ham BCTs Credible source Social support (unspecified) Social support (practical) Social support (emotional) Remove adverse stimulus	Ethnic background Delivery of education session in various languages by GP and heath care assistant who were able to communicate in a variety of different local Languages (Gujarati, Urdu, Punjabi). Interpreters involved in processing of written informed consent during screening at faith centres BCTs Social Support (practical) Credible Source Restructuring the social environment
Adaptations of program content: Tailoring of language, visuals, examples, scenarios, and activities used during the intervention And Cognitive adaptation intervention—Information processing characteristics such as language and age/developmental level		Intervention content/activities tailored to address local needs identified from stakeholder engagement meetings. Example of activities included social gathering that included healthy food, gardening, stress management groups, involvement in leadership and co-ordination, improving physical environments, such as direct involvement of local police in providing safer spaces BCTs Action planning Demonstration of a behaviour Framing/reframing Restructuring the physical environment Restructuring the social environment			Intervention content/activities The video contained information in concordance with the guidelines promoted by British Heart Foundation, but with a focus towards the Bangladeshi culture. The content designed with consideration for target audience's culture, taboo topics BCTs Information about health consequences	Intervention content/activities Participants were able to access additional PA sessions at different times of the week, delivered by the same provider (NUR FITNESS CIC) Language/visuals Bespoke dietary advice tailored to participants BCTs Social Support Practical Instruction on how to perform the behaviour 12.1 Restructuring the physical environment	Intervention content/activities Dietitians modified traditional high-calorie dishes to meet nutritional recommendations while retaining original appearance, flavour and textures Pedometers given to the participants to provide step counts for motivation through self-monitoring and for progress assessments A South Asian recipe book; Bollywood style drama DVD on the prevention of diabetes; South Asian Balance of Good Health food placement Language/visuals etc “The dietitians’ toolkit” adapted and translated health promotion resources in English, Urdu, Hindi and Gurmukhi (written Punjabi) Leaflets on eating and physical activity: Pictorial and written examples of physical activity e.g. yoga, Bollywood dancing and people in South Asian clothes etc Counterweight leaflet adapted by swapping some ‘Western foods’ like minced beef lasagne with traditional South Asian dishes e.g., chapati with lamb and spinach Traditional dishes prepared at home photographed in small, medium and large portions to guide participants in reporting their dietary intake Patient information leaflets—Information on diabetes, IGT/IFG and the trial changed from literal to spoken language, e.g., diabetes also called sugar disease. Translated into Urdu, Gurmukhi BCTs Discrepancy between current and behavioural goal Self-monitoring of behaviour Biofeedback (pedometers) Feedback on outcome of behaviour Framing / reframing Instruction on how to perform a behaviour Credible source Restructuring the social environment Adding objects to the environment	
Adaptations of program form effectiveness: Altering program structure and goals, which have a potential to reduce program effectiveness	Intervention structure Advance completion of aspects of the intervention (i.e., lifestyle questions on physical activity, smoking and alcohol) with the aim of saving time during the face-to face health check appointment. Signposting people, where appropriate, to local lifestyle services, based on responses to the lifestyle questions BCTs Information about health consequences Prompts cues						Intervention outcomes Changed to more achievable targets e.g., Weight loss goals: 2.5 kg more in the intervention than the control group (instead of 5%) Physical activity: Moderate intensity > 30 min/day. Emphasis on walking (instead of supervised physical activity training sessions) Dietary targets—not specific to nutrients but focused on cooking methods, portion size, food choices, amount of fat used in cooking and encouraging foods high in dietary fibre BCTs Goal setting (outcome) Information on how to perform behaviour	

BCTs = Behaviour Change Techniques

### Affective-Motivational Adaptation and Cultural Adaptations

Affective-motivational adaptation and cultural adaptations were the most frequently reported dimensions of the adaptation framework. Cultural adaptations are defined as tailoring the intervention to meet a community’s worldview and lifestyle. Affective-motivational adaptations include those related to gender, ethnic background, values, traditions, religious background, and socioeconomic status. Adaptations in these categories primarily utilised strategies such as using credible sources, social support and/or restructuring the social environment. For example, five of the nine reported interventions matched participants ethnic background, culture, traditions and language with that of the intervention provider. Other adaptations considered religious and cultural backgrounds by deleting foods on information leaflets deemed as taboo to Muslims (e.g., pork and ham) [80, 81] and providing women only intervention sessions [74]. The family influence in South Asian families was integrated by providing social support in the form of recruiting family clusters, specifically the family cook, instead of targeting just the individual. Two interventions, one of which was conducted in a deprived area, also considered participants’ socio-economic status by providing a material incentive in the form of free or subsidized access to intervention activities.

### Environmental Adaptations [53, 57, 60, 64, 69–72]

Seven interventions included environmental adaptations which relate to ecological aspects of the community primarily aimed at restructuring the physical and social environment. Adaptations included intervention delivery in venues which were local, convenient, and familiar (e.g., community halls). Other adaptations restructured the social environment by intervention delivery in venues associated with participants values and beliefs (e.g., religious venues). Social support was provided by credible sources which participants viewed as non-judgemental (e.g., home or non-gym settings). One intervention [67] used prompts/cues as a strategy of recruitment prioritising telephone over postal communication.

### Cognitive Adaptations and Adaptations of Program Content [53, 59, 64, 69–72]

Adaptations of program content involve the adapting visuals, examples, scenarios, and activities. Cognitive adaptations involved tailoring information processing characteristics such as language and age/developmental level. Five studies included cognitive and program content adaptations, with the PODOSA trial reporting the most adaptations**.** These adaptations included 1) modification of participants traditional high-calorie dishes to meet nutritional recommendations while retaining their original appearance, flavour and textures; 2) modification of information leaflets to match participants spoken language; 3) tailoring pictorial and written examples of physical activity and diet leaflets (e.g. Swapping ‘Western foods’ with traditional South Asian dishes such as chapati with lamb and spinach); 4) tailoring patient information leaflets on diabetes from literal to spoken language, (e.g., diabetes also called sugar disease). The strategies behind these modifications primarily worked to highlight the discrepancy between current and behavioural goal, provide participants with instructions on how to perform a behaviour and provide information about health consequences from a credible source by restructuring the social environment and adding objects to the environment e.g., pedometers.

### Adaptations of Program Form Effectiveness [67, 80, 81]

Adaptations of program form effectiveness involves altering the program structure and goals, which have a potential to reduce program effectiveness. The three-year PODOSA intervention modified the goals of the intervention by making weight loss, physical and dietary targets more achievable (from 5 to 3kgs). In this study, lowering the weight loss target led to modest but sustained mean (SD) weight loss in the intervention group (Mean 1·13 kg (SD 4·12), compared with a mean weight gain of 0·51 kg (3·65) in the control group. The Telephone outreach intervention modified the structure of the NHS Health Check intervention by completion of part of the intervention during the recruitment call and signposting of people, where appropriate, to local lifestyle services, based on responses to the lifestyle questions. However, this modification though successful in increasing intervention uptake, had a small influence of modifying behaviours related to diet and physical activity.

## Discussion

This review reports the extent to which barriers, enablers and culturally adapted lifestyle interventions (diet or physical activity) intended to prevent or delay the onset of T2D and related conditions (i.e., CVD and Obesity) have been explored in UK minority ethnic populations. This review was conducted in the context of low uptake and completion rates amongst minority ethnic groups in English nationwide interventions for preventing diabetes and CVD[19, 32, 33, 37]. The findings show that in the recent decade, studies in this population have largely focused on exploring barriers and enablers, rather than culturally adapting interventions [76, 78].

Our scoping review has highlighted barriers and enablers related to social opportunity (i.e., social norms and cues that can encourage or discourage behaviour change) and reflective motivation (high cognitive processes, such as beliefs, values and goals)[50], as strong determinants of behaviour change for UK minority ethnic groups. Our findings align with previous UK research which identified social norms and values in South Asian, African and Caribbean communities’ cultures as important enablers to engagement in lifestyle and self-management programmes [83, 84]. For example Moore et al., reporting the development of a culturally sensitive self-management support programme for people diagnosed with T2D for UK African and Caribbean communities [84], also found that even in the presence of adequate levels of knowledge, motivation to perform healthy diabetes-related self-management behaviours may be limited by specific cultural beliefs and cultural/social norms. The integral role and influence of social and cultural experiences and beliefs amongst South Asian, African and African Caribbean communities therefore needs adequate consideration when tailoring interventions for UK minority ethnic populations.

Our scoping review has highlighted three key strategies for addressing social and cultural barriers to engagement for minority ethnic groups including: using credible sources (e.g., matching participants ethnic background and language with that of the intervention provider); providing social support (e.g., family, peers, mentors); and restructuring the social environment (e.g., deleting foods on information leaflets deemed as taboo to Muslims). Such strategies aim to meet the community’s worldview and lifestyle as well as consider participants gender, ethnic background, values, traditions, religious background, and socioeconomic status[51]. Our findings reflect those of a 2024 systematic review, which although focused on diabetes prevention programmes with no geographical restrictions, also highlighted social support and the implementation of culturally appropriate programmes that considered specific needs, values and preferences of diverse populations as the most common mechanisms that increase engagement [85].

A key recommendation from this review would therefore be for UK interventions aimed at preventing T2D and related conditions to incorporate engagement strategies aimed at both addressing the highlighted social and cultural barriers. A 2023 narrative review of evidence from the NHS DPP highlighted that despite existing service specifications recommending adapting intervention to local population, there were still shortfalls in addressing the needs of diverse populations, including minority ethnic groups [86]. A separate qualitative evaluation described current efforts of adapting the programme, which largely focused on providing developed course materials in different languages, as inadequate for increasing equity of access. Although our scoping review has identified a few barriers within psychological capability (e.g., language and health literacy) and enablers (e.g., dietary advice accommodating a more diverse and international food diet), there is a strong indication that strategies to address barriers related to social and cultural aspects are stronger determinants of behaviour change for UK minority ethnic groups even in the presence of adequate knowledge[84].

Physical opportunity, which encompasses environmental cues and resources, such as time or money, has also been identified as an important determinant for participation among UK ethnic minorities. Our previous work undertaken in a largely White British population, indicated physical opportunity (i.e., convenient location and flexible intervention session times e.g., evening classes) to be the most important determinant for engagement in the NHS DPP [87]. However, rather than focusing on location proximity, the findings of this review emphasise that for minority ethnic groups, strategies that focus on other aspects of physical opportunity such as culturally acceptable, trusted and safe venues and locations are more important for positive behaviour change. In addition, the design of such strategies would need input from community or religious leaders/champions with adequate understanding of settings considered as a safe and culturally acceptable.

Our scoping review has also highlighted the importance of considering socio-economic positioning when developing interventions for minority ethnic groups in the UK. This resonates with previous research which has highlighted the relationship between ethnicity and socioeconomic status [88, 89]. Highlighted strategies resonates with other research which suggest using adaptations to address economic barriers and financial constraints including the provision of incentives such as free/affordable programmes and monetary rewards [85]. However, implementing such strategies is a UK context where programmes such as the NHS DPP and NHS Health checks are already nationally funded, would need to utilise different approaches e.g., providing food vouchers to support maintenance of healthy diets.

Finally, an important finding in this review is the need to consider the role of social experiences (e.g., racism and discrimination) that foster mistrust and lead to disengagement, in efforts to increase uptake in lifestyle intervention for minority ethnic groups. This scoping review suggest using more person-centered strategies to intervention delivery, which aim to build trust and understanding, could be vital for continued engagement. However, more work needs to be done to explore how this could be delivered within context that largely implement group-based interventions.

### Strengths and Limitations

This is the first scoping review to identify barriers and enablers to uptake and engagement in diet and physical activity interventions for UK minority ethnic groups and consider potential strategies for cultural adaptations. The review provides recommendations for strategies to promote uptake, based on empirical evidence and theoretical underpinning. The incorporation of behaviour change theory provides a base for developing culturally tailored interventions for preventing T2D and related conditions in UK ethnic minority populations. The findings of this review, although primarily focusing on the UK setting, could be relevant for consideration in other high income European countries where DPPs are provided (e.g., Finland) including. A limitation of the study is the low representation African and African Caribbean populations in the included studies. Ethnic minority groups comprise 18% of the UK population, with Asian ethnic groups comprising 9.3% of the population followed by Black ethnic groups (4.0%) [90]. Most studies included in this scoping review have been conducted in South Asian populations, highlighting limited evidence for African and African Caribbean populations [83]. In addition, most research to date has focused on English cities with the greatest diversity (i.e., London, Leicester, Manchester)[91]. However, with recent [91] expansion of ethnic communities across other regions in England and Wales [91], it is important to ensure that research exploring diabetes prevention extends beyond these cities to many formerly non-diverse regions and also targets other high risk groups including Black African and African Caribbean ethnic groups [83].

## Conclusion

A theory-informed examination of barriers and enablers to engagement in lifestyle interventions amongst UK minority ethnic groups has identified the central role of family, culture, beliefs, and socio-economic circumstances in determining behaviour change. The findings have identified the most impactful strategies for behaviour change as those providing information from credible sources and social support as well as restructuring the social and physical environment. This can be implemented by delivering interventions in environments considered by participants to be local, convenient, safe and culturally acceptable. Important research gaps include investigating tailored prevention interventions for African and African Caribbean populations and exploration of the influence of negative social experiences e.g., racism and prejudice on engagement with diabetes prevention interventions.

## References

[1] Zheng Y, Ley SH, Hu FB. “Global aetiology and epidemiology of type 2 diabetes mellitus and its complications,” (in eng). Nat Rev Endocrinol. 2018;14(2):88–98. 10.1038/nrendo.2017.151. 29219149 10.1038/nrendo.2017.151

[2] NCD Risk Factor Collaboration (NCD-RisC), "Worldwide trends in diabetes since 1980: a pooled analysis of 751 population-based studies with 4.4 million participants," (in eng),. Lancet 387, 10027 1513–1530 2016 10.1016/s0140-6736(16)00618-8 10.1016/S0140-6736(16)00618-8 PMC5081106 27061677

[3] Cho NH, et al. “IDF Diabetes Atlas: Global estimates of diabetes prevalence for 2017 and projections for 2045,” (in eng). Diabetes Res Clin Pract. 2018;138:271–81. 10.1016/j.diabres.2018.02.023. 29496507 10.1016/j.diabres.2018.02.023

[4] Diabetes UK. "Diabetes Statistics." https://www.diabetes.org.uk/professionals/position-statements-reports/statistics (accessed 2nd of August, 2023)

[5] Hex N, Bartlett C, Wright D, Taylor M, Varley D. “Estimating the current and future costs of Type 1 and Type 2 diabetes in the UK, including direct health costs and indirect societal and productivity costs,” (in eng). Diabet Med. 2012;29(7):855–62. 10.1111/j.1464-5491.2012.03698.x. 22537247 10.1111/j.1464-5491.2012.03698.x

[6] OpenPrescribing. High-level prescribing trends for Antidiabetic drugs (BNF section 6.1.2) across all GP practices in NHS England for the last five years. https://openprescribing.net/bnf/060102/. Accessed 12 July 2023.

[7] Goff LM. “Ethnicity and Type 2 diabetes in the UK,” (in eng). Diabet Med. 2019;36(8):927–38. 10.1111/dme.13895. 30614072 10.1111/dme.13895

[8] Pham TM, Carpenter JR, Morris TP, Sharma M, Petersen I. “Ethnic Differences in the Prevalence of Type 2 Diabetes Diagnoses in the UK: Cross-Sectional Analysis of the Health Improvement Network Primary Care Database,” (in eng). Clin Epidemiol. 2019;11:1081–8. 10.2147/clep.S227621. 32021464 10.2147/CLEP.S227621 PMC6948201

[9] Y. Chen et al., "Relationship between body composition indicators and risk of type 2 diabetes mellitus in Chinese adults," BMC Public Health, 20 1, 452, 2020/04/06 2020, 10.1186/s12889-020-08552-5 10.1186/s12889-020-08552-5 PMC7137510 32252701

[10] I. Kyrou et al., "Sociodemographic and lifestyle-related risk factors for identifying vulnerable groups for type 2 diabetes: a narrative review with emphasis on data from Europe," (in eng),. BMC Endocr Disord 20 Suppl 1, 134, 2020, 10.1186/s12902-019-0463-3 10.1186/s12902-019-0463-3 PMC7066728 32164656

[11] National Institute for Health and Care Excellence. Diabetes - type 2: What are the risk factors?. 2024. https://cks.nice.org.uk/topics/diabetes-type-2/background-information/risk-factors/. Accessed 9 Aug 2024.

[12] GOV.UK. "Obesity Profile: short statistical commentary May 2023." https://www.gov.uk/government/statistics/obesity-profile-update-may-2023/obesity-profile-short-statistical-commentary-may-2023 (accessed 19 June, 2024)

[13] Kyrou I, Randeva H S, Tsigos C, Kaltsas G, Weickert MO. Clinical Problems Caused by Obesity. 2018. https://www.ncbi.nlm.nih.gov/books/NBK278973/. Accessed 19 June 2024.

[14] Misra A, Khurana L. “Obesity-related non-communicable diseases: South Asians vs White Caucasians,” (in eng). Int J Obes (Lond). 2011;35(2):167–87. 10.1038/ijo.2010.135. 20644557 10.1038/ijo.2010.135

[15] Wells JC. “Ethnic variability in adiposity, thrifty phenotypes and cardiometabolic risk: addressing the full range of ethnicity, including those of mixed ethnicity,” (in eng). Obes Rev. 2012;13(Suppl 2):14–29. 10.1111/j.1467-789X.2012.01034.x. 23107256 10.1111/j.1467-789X.2012.01034.x

[16] Meeks KA, et al. “Disparities in type 2 diabetes prevalence among ethnic minority groups resident in Europe: a systematic review and meta-analysis,” (in eng). Intern Emerg Med. 2016;11(3):327–40. 10.1007/s11739-015-1302-9. 26370238 10.1007/s11739-015-1302-9

[17] Oldroyd J, Banerjee M, Heald A, Cruickshank K. “Diabetes and ethnic minorities,” (in eng). Postgrad Med J. 2005;81(958):486–90. 10.1136/pgmj.2004.029124. 16085737 10.1136/pgmj.2004.029124 PMC1743339

[18] Leon BM, Maddox TM. “Diabetes and cardiovascular disease: Epidemiology, biological mechanisms, treatment recommendations and future research,” (in eng). World J Diabetes. 2015;6(13):1246–58. 10.4239/wjd.v6.i13.1246. 26468341 10.4239/wjd.v6.i13.1246 PMC4600176

[19] TR Einarson, A Acs, C Ludwig, UH Panton "Prevalence of cardiovascular disease in type 2 diabetes: a systematic literature review of scientific evidence from across the world in 2007–2017,". Cardiovasc Diabetol, 17 1 83 2018, 10.1186/s12933-018-0728-6 10.1186/s12933-018-0728-6 PMC5994068 29884191

[20] National Health Service. The NHS Long Term Plan. 2019. [Online]. Available: https://www.longtermplan.nhs.uk/wp-content/uploads/2019/08/nhs-long-term-plan-version-1.2.pdf. Accessed 20 June 2024.

[21] NHS England. NHS Diabetes Prevention Programme (NHS DPP) [Online]. https://www.england.nhs.uk/diabetes/diabetes-prevention/. Accessed 3 Nov 2022.

[22] Saaristo T, et al. “Lifestyle intervention for prevention of type 2 diabetes in primary health care: one-year follow-up of the Finnish National Diabetes Prevention Program (FIN-D2D),” (in eng). Diabetes Care. 2010;33(10):2146–51. 10.2337/dc10-0410. 20664020 10.2337/dc10-0410 PMC2945150

[23] Lindström J, et al. “The Finnish Diabetes Prevention Study (DPS): Lifestyle intervention and 3-year results on diet and physical activity,” (in eng). Diabetes Care. 2003;26(12):3230–6. 10.2337/diacare.26.12.3230. 14633807 10.2337/diacare.26.12.3230

[24] Thankappan KR, et al. “A peer-support lifestyle intervention for preventing type 2 diabetes in India: A cluster-randomized controlled trial of the Kerala Diabetes Prevention Program,” (in eng). PLoS Med. 2018;15(6):e1002575. 10.1371/journal.pmed.1002575. 29874236 10.1371/journal.pmed.1002575 PMC5991386

[25] Ramachandran A, Snehalatha C, Mary S, Mukesh B, Bhaskar AD, Vijay V. “The Indian Diabetes Prevention Programme shows that lifestyle modification and metformin prevent type 2 diabetes in Asian Indian subjects with impaired glucose tolerance (IDPP-1),” (in eng). Diabetologia. 2006;49(2):289–97. 10.1007/s00125-005-0097-z. 16391903 10.1007/s00125-005-0097-z

[26] Pan XR, et al. “Effects of diet and exercise in preventing NIDDM in people with impaired glucose tolerance. The Da Qing IGT and Diabetes Study,” (in eng). Diabetes Care. 1997;20(4):537–44. 10.2337/diacare.20.4.537. 9096977 10.2337/diacare.20.4.537

[27] Ely EK, et al. “A National Effort to Prevent Type 2 Diabetes: Participant-Level Evaluation of CDC’s National Diabetes Prevention Program,” (in eng). Diabetes Care. 2017;40(10):1331–41. 10.2337/dc16-2099. 28500215 10.2337/dc16-2099 PMC5606310

[28] Knowler WC, et al. “Reduction in the incidence of type 2 diabetes with lifestyle intervention or metformin,” (in eng). N Engl J Med. 2002;346(6):393–403. 10.1056/NEJMoa012512. 11832527 10.1056/NEJMoa012512 PMC1370926

[29] Dunbar JA, et al. Scaling Up Diabetes Prevention in Victoria, Australia: Policy Development, Implementation, and Evaluation. Diabetes Care. 2014;37(4):934–42. 10.2337/dc12-2647. 24319121 10.2337/dc12-2647

[30] McManus E, Meacock R, Parkinson B, Sutton M. “Population level impact of the NHS Diabetes Prevention Programme on incidence of type 2 diabetes in England: An observational study,” (in eng). Lancet Reg Health Eur. 2022;19:100420. 10.1016/j.lanepe.2022.100420. 35664052 10.1016/j.lanepe.2022.100420 PMC9160476

[31] Valabhji J, et al. “Early Outcomes From the English National Health Service Diabetes Prevention Programme,” (in eng). Diabetes Care. 2020;43(1):152–60. 10.2337/dc19-1425. 31719054 10.2337/dc19-1425 PMC7115827

[32] Whelan M, Bell L. “The English national health service diabetes prevention programme (NHS DPP): A scoping review of existing evidence,” (in eng). Diabet Med. 2022;39(7):e14855. 10.1111/dme.14855. 35441747 10.1111/dme.14855 PMC9321029

[33] Howarth E, et al. ‘Going the distance’: an independent cohort study of engagement and dropout among the first 100 000 referrals into a large-scale diabetes prevention program. BMJ Open Diabetes Res Care. 2020;8(2):e001835. 10.1136/bmjdrc-2020-001835. 33303493 10.1136/bmjdrc-2020-001835 PMC7733095

[34] Usher-Smith J, Mant J, and MA. NHS health check programme rapid evidence synthesis. University of Cambridge. 2017. file://ueahome/eressci24/w0449075/data/Downloads/NHS%20Health%20Check%20Report%20Final.pdf. Accessed 20 June 2024.

[35] National Health Service. "NHS health checks " https://www.england.nhs.uk/ltphimenu/cvd/nhs-health-checks/Google (accessed 19 June, 2024)

[36] Tanner L, Kenny RPW, Still M, Pearson F, and Bhardwaj-Gosling F, "NHS Health Check Programme Rapid Review Update," University of Sunderland and Newcastle University 2020, 2020

[37] Molokhia M, et al. “What factors influence differential uptake of NHS Health Checks, diabetes and hypertension reviews among women in ethnically diverse South London? Cross-sectional analysis of 63,000 primary care records,” (in eng). EClinicalMedicine. 2022;49:101471. 10.1016/j.eclinm.2022.101471. 35747176 10.1016/j.eclinm.2022.101471 PMC9156982

[38] National Institute for Health and Care Excellence. Type 2 diabetes: prevention in people at high risk. 2017. [Online]. Available: https://www.nice.org.uk/guidance/ph38/resources/type-2-diabetes-prevention-in-people-at-high-risk-pdf-1996304192197. Accessed 20 June 2024.

[39] NHS England. NHS DPP Service Specifcation 2022. 2022. [Online]. Available: https://www.england.nhs.uk/wp-content/uploads/2016/08/Diabetes-Prevention-Programme-Framework-3-Service-Specification-October-2022.pdf. Accessed 20 June 2024.

[40] Gov.UK. "Preventing illness and improving health for all: a review of the NHS Health Check programme and recommendations." https://www.gov.uk/government/publications/nhs-health-check-programme-review/preventing-illness-and-improving-health-for-all-a-review-of-the-nhs-health-check-programme-and-recommendations#the-reviews-6-recommendations (accessed 19 June, 2024)

[41] Lagisetty PA, et al. “Culturally Targeted Strategies for Diabetes Prevention in Minority Population,” (in eng). Diabetes Educ. 2017;43(1):54–77. 10.1177/0145721716683811. 28118127 10.1177/0145721716683811 PMC5408505

[42] Sarte AF, Fong M, Yung K, Ng L, Koehn S, Sohal P. Culturally-Appropriate Prediabetes Lifestyle Intervention Programs: a Review of the Literature. Can J Diabetes. 2012;36(5):S33–4. 10.1016/j.jcjd.2012.07.320.

[43] McCurley JL, Gutierrez AP, Gallo LC. “Diabetes Prevention in U.S. Hispanic Adults: a Systematic Review of Culturally Tailored Interventions,” (in eng). Am J Prev Med. 2017;52(4):519–29. 10.1016/j.amepre.2016.10.028. 27989451 10.1016/j.amepre.2016.10.028 PMC5362335

[44] Ali MR, Nacer H, Lawson CA, Khunti K. “Racial and Ethnic Disparities in Primary Prevention of Cardiovascular Disease,” (in eng). Can J Cardiol. 2024;40(6):1016–30. 10.1016/j.cjca.2024.01.028. 38309463 10.1016/j.cjca.2024.01.028

[45] Sanders Thompson VL, Johnson-Jennings M, Bauman AA, Proctor E. “Use of culturally focused theoretical frameworks for adapting diabetes prevention programs: a qualitative review,” (in eng). Prev Chronic Dis. 2015;12:E60. 10.5888/pcd12.140421. 25950567 10.5888/pcd12.140421 PMC4436044

[46] Ali SH, Misra S, Parekh N, Murphy B, DiClemente RJ. “Preventing type 2 diabetes among South Asian Americans through community-based lifestyle interventions: A systematic review,” (in eng). Prev Med Rep. 2020;20:101182. 10.1016/j.pmedr.2020.101182. 32844084 10.1016/j.pmedr.2020.101182 PMC7441043

[47] Wadi NM, Asantewa-Ampaduh S, Rivas C, Goff LM. “Culturally tailored lifestyle interventions for the prevention and management of type 2 diabetes in adults of Black African ancestry: a systematic review of tailoring methods and their effectiveness,” (in eng). Public Health Nutr. 2022;25(2):422–36. 10.1017/s1368980021003682. 34435943 10.1017/S1368980021003682 PMC8883766

[48] Nieto-Martínez R, González-Rivas JP, Aschner P, Barengo NC, Mechanick JI. Transculturalizing Diabetes Prevention in Latin America. Ann Glob Health. 2017;83(3):432–43. 10.1016/j.aogh.2017.07.001. 29221516 10.1016/j.aogh.2017.07.001

[49] Montesi L, Caletti MT, Marchesini G. “Diabetes in migrants and ethnic minorities in a changing World,” (in eng). World J Diabetes. 2016;7(3):34–44. 10.4239/wjd.v7.i3.34. 26862371 10.4239/wjd.v7.i3.34 PMC4733447

[50] Michie S, Atkins L, West R. The Behaviour Change Wheel: A Guide to Designing Interventions. London: Silverback; 2014.

[51] Castro FG, Barrera M, Martinez CR. The Cultural Adaptation of Prevention Interventions: Resolving Tensions Between Fidelity and Fit. Prev Sci. 2004;5(1):41–5. 10.1023/B:PREV.0000013980.12412.cd. 15058911 10.1023/b:prev.0000013980.12412.cd

[52] Michie S, et al. “The behavior change technique taxonomy (v1) of 93 hierarchically clustered techniques: building an international consensus for the reporting of behavior change interventions,” (in eng). Ann Behav Med. 2013;46(1):81–95. 10.1007/s12160-013-9486-6. 23512568 10.1007/s12160-013-9486-6

[53] Tricco AC, et al. “PRISMA Extension for Scoping Reviews (PRISMA-ScR): Checklist and Explanation,” (in eng). Ann Intern Med. 2018;169(7):467–73. 10.7326/m18-0850. 30178033 10.7326/M18-0850

[54] Arksey H, O’Malley L. Scoping Studies: Towards a Methodological Framework. Int J Soc Res Methodol: Theory Pract. 2005;8(1):19–32. 10.1080/1364557032000119616.

[55] GOV.UK. Ethnicity facts and figures: Writing about ethnicity. https://www.ethnicity-facts-figures.service.gov.uk/style-guide/writing-about-ethnicity. Accessed 14 Nov 2022.

[56] National Institute for Health and Care Excellence. Type 2 diabetes: prevention in people at high risk. 2012. [Online]. Available: https://www.nice.org.uk/guidance/ph38. Accessed 20 June 2024.

[57] Miles LM, Hawkes RE, French DP. “How is the Behavior Change Technique Content of the NHS Diabetes Prevention Program Understood by Participants? A Qualitative Study of Fidelity, With a Focus on Receipt,” (in eng). Ann Behav Med. 2022;56(7):749–59. 10.1093/abm/kaab093. 34788358 10.1093/abm/kaab093 PMC9274983

[58] Kumpfer KL, Alvarado R, Smith P, Bellamy N. Cultural Sensitivity and Adaptation in Family-Based Prevention Interventions. Prev Sci. 2002;3(3):241–6. 10.1023/A:1019902902119. 12387558 10.1023/a:1019902902119

[59] Michie S, van Stralen MM, West R. The behaviour change wheel: a new method for characterising and designing behaviour change interventions. Implement Sci. 2011;6(1):42. 10.1186/1748-5908-6-42. 21513547 10.1186/1748-5908-6-42 PMC3096582

[60] Bhatnagar P, Foster C, Shaw A. “Barriers and facilitators to physical activity in second-generation British Indian women: A qualitative study,” (in eng). PLoS One. 2021;16:11. 10.1371/journal.pone.0259248. 10.1371/journal.pone.0259248 PMC8565737 34731201

[61] Curry WB, Duda JL, Thompson JL. “Perceived and Objectively Measured Physical Activity and Sedentary Time among South Asian Women in the UK,” (in eng). Int J Environ Res Public Health. 2015;12(3):3152–73. 10.3390/ijerph120303152. 25785499 10.3390/ijerph120303152 PMC4377957

[62] Emadian A, Thompson JL. “A Mixed-Methods Examination of Physical Activity and Sedentary Time in Overweight and Obese South Asian Men Living in the United Kingdom,” (in eng). Int J Environ Res Public Health. 2017;14:4. 10.3390/ijerph14040348. 10.3390/ijerph14040348 PMC5409549 28346386

[63] A Garner-Purkis S Alageel C Burgess, M Gulliford, "A community-based, sport-led programme to increase physical activity in an area of deprivation: a qualitative case study," (in eng), BMC Public Health, 20, 2020, 10.1186/s12889-020-08661-1 10.1186/s12889-020-08661-1 PMC7322853 32600289

[64] Horne M, Emsley R, Woodham A, Wearden A, Skelton DA. “Associations of intention to undertake physical activity among community dwelling British South Asian adults aged 60 years and over: a cross-sectional study,” (in English). Public Health. 2018;162:1–8. 10.1016/j.puhe.2018.05.005. 29913349 10.1016/j.puhe.2018.05.005

[65] Horne M, Skelton DA, Speed S, Todd C. “Perceived barriers to initiating and maintaining physical activity among South Asian and White British adults in their 60s living in the United Kingdom: a qualitative study,” (in English). Ethn Health. 2013;18(6):626–45. 10.1080/13557858.2013.814762. 23834070 10.1080/13557858.2013.814762

[66] Emadian A, England CY, Thompson JL. “Dietary intake and factors influencing eating behaviours in overweight and obese South Asian men living in the UK: mixed method study,” (in eng). BMJ Open. 2017;7:7. 10.1136/bmjopen-2017-016919. 10.1136/bmjopen-2017-016919 PMC5541587 28729327

[67] Brangan E, Stone TJ, Chappell A, Harrison V, Horwood J. “Patient experiences of telephone outreach to enhance uptake of NHS Health Checks in more deprived communities and minority ethnic groups: A qualitative interview study,” (in English). Health Expectations : Int J Public Participation Health Care Health Policy. 2019;22(3):364–72. 10.1111/hex.12856. 10.1111/hex.12856 PMC6543263 30585389

[68] L Cross-Bardell, T George, M Bhoday, H Tuomainen, N Qureshi, J. Kai, "Perspectives on enhancing physical activity and diet for health promotion among at-risk urban UK South Asian communities: a qualitative study," (in eng), BMJ Open, 5 ;2015; 2 10.1136/bmjopen-2014-007317 10.1136/bmjopen-2014-007317 PMC4346672 25724983

[69] Derges J, et al. “Well London” and the benefits of participation: results of a qualitative study nested in a cluster randomised trial. BMJ Open. 2014;4(4):e003596. 10.1136/bmjopen-2013-003596. 24694622 10.1136/bmjopen-2013-003596 PMC3987724

[70] Eastwood SV, Rait G, Bhattacharyya M, Nair DR, Walters K. Cardiovascular risk assessment of South Asian populations in religious and community settings: a qualitative study. Fam Pract. 2013;30(4):466–72. 10.1093/fampra/cmt017. 23629737 10.1093/fampra/cmt017

[71] Liljas AEM, et al. Engaging ‘hard to reach’ groups in health promotion: the views of older people and professionals from a qualitative study in England. BMC Public Health. 2019;19(1):629. 10.1186/s12889-019-6911-1. 31122239 10.1186/s12889-019-6911-1 PMC6533740

[72] Ochieng BM. “Black African migrants: the barriers with accessing and utilizing health promotion services in the UK,” (in eng). Eur J Public Health. 2012;23(2):265–9. 10.1093/eurpub/cks063. 22683768 10.1093/eurpub/cks063

[73] Ochieng BMN. “Black families’ perceptions of barriers to the practice of a healthy lifestyle: A qualitative study in the UK,” (in English). Critical Public Health. 2013;23(1):6–16. 10.1080/09581596.2011.610438.

[74] Penn L, Dombrowski SU, Sniehotta FF, White M. “Perspectives of UK Pakistani women on their behaviour change to prevent type 2 diabetes: qualitative study using the theory domain framework,” (in eng). BMJ Open. 2014;4:7. 10.1136/bmjopen-2013-004530. 10.1136/bmjopen-2013-004530 PMC4091164 25005595

[75] Tomalin E, Sadgrove J, Summers R. Health, faith and therapeutic landscapes: Places of worship as Black, Asian and Minority Ethnic (BAME) public health settings in the United Kingdom. Soc Sci Med. 2019;1982(230):57–65. 10.1016/j.socscimed.2019.03.006. 10.1016/j.socscimed.2019.03.006 30965184

[76] Barry E, Greenhalgh T. “How do UK general practice staff understand and manage prediabetes? A focus group study,” (in English). BJGP Open. 2022;6(2):BJGO.2021.0166. 10.3399/BJGPO.2021.0166. 10.3399/BJGPO.2021.0166 PMC9447313 35523431

[77] Morrison Z, Douglas A, Bhopal R, Sheikh A, Trial I. Understanding experiences of participating in a weight loss lifestyle intervention trial: a qualitative evaluation of South Asians at high risk of diabetes. BMJ Open. 2014;4(6):e004736. 10.1136/bmjopen-2013-004736. 24951108 10.1136/bmjopen-2013-004736 PMC4067864

[78] Twohig H, Hodges V, Hobbis C, Mitchell C. “Response to diagnosis of pre-diabetes in socioeconomically deprived areas: a qualitative study,” (in eng). BJGP Open. 2019;3:3. 10.3399/bjgpopen19X101661. 10.3399/bjgpopen19X101661 PMC6970589 31581115

[79] Latif S, Ahmed I, Amin MS, Syed I, Ahmede N. “Exploring the potential impact of health promotion videos as a low cost intervention to reduce health inequalities: A pilot before and after study on Bangladeshis in Inner-city London,” (in English). London J Primary Care. 2016;8(4):66–71. 10.1080/17571472.2016.1208382. 10.1080/17571472.2016.1208382 PMC5330360 28250836

[80] Wallia S, et al. “Culturally adapting the prevention of diabetes and obesity in South Asians (PODOSA) trial,” (in English). Health Promot Int. 2013;29(4):768–79. 10.1093/heapro/dat015. 23574693 10.1093/heapro/dat015

[81] Bhopal RS, et al. “Effect of a lifestyle intervention on weight change in south Asian individuals in the UK at high risk of type 2 diabetes: A family-cluster randomised controlled trial,” (in English). Lancet Diabetes Endocrinol. 2014;2(3):218–27. 10.1016/s2213-8587(13)70204-3. 24622752 10.1016/S2213-8587(13)70204-3

[82] Willis A, et al. “A community faith centre based screening and educational intervention to reduce the risk of type 2 diabetes: A feasibility study,” (in eng). Diabetes Res Clin Pract. 2016;120:73–80. 10.1016/j.diabres.2016.07.025. 27522562 10.1016/j.diabres.2016.07.025

[83] Patel N, et al. “Barriers and Facilitators to Healthy Lifestyle Changes in Minority Ethnic Populations in the UK: a Narrative Review,” (in eng). J Racial Ethn Health Disparities. 2017;4(6):1107–19. 10.1007/s40615-016-0316-y. 27928772 10.1007/s40615-016-0316-y PMC5705764

[84] Moore AP, Rivas CA, Stanton-Fay S, Harding S, Goff LM. Designing the Healthy Eating and Active Lifestyles for Diabetes (HEAL-D) self-management and support programme for UK African and Caribbean communities: a culturally tailored, complex intervention under-pinned by behaviour change theory. BMC Public Health. 2019;19(1):1146. 10.1186/s12889-019-7411-z. 31429735 10.1186/s12889-019-7411-z PMC6702734

[85] B McMullen, K Duncanson, C Collins, L MacDonald-Wicks, "A systematic review of the mechanisms influencing engagement in diabetes prevention programmes for people with pre-diabetes," (in eng),. Diabet Med e15323, 2024; 10.1111/dme.15323 10.1111/dme.15323 38829966

[86] C. Koning, M. Pelletier, and J. Spooner, "The national health service England diabetes prevention program—A narrative review," J Diabetol 14, 4 2023. [Online]. Available: https://journals.lww.com/jodb/fulltext/2023/14040/the_national_health_service_england_diabetes.4.aspx

[87] Katangwe T, Family H, Sokhi J, Kirkdale CL, Twigg MJ. The community pharmacy setting for diabetes prevention: A mixed methods study in people with ‘pre-diabetes.’ Res Soc Adm Pharm. 2020;16(8):1067–80. 10.1016/j.sapharm.2019.11.001. 10.1016/j.sapharm.2019.11.001 31734102

[88] U.S. Census Bureau, "American Indian/Alaska Native, Hispanic, Pacific Islander and Native Hawaiian families are more likely than Caucasian and Asian families to live in poverty," 2014

[89] American Psychological Association. Ethnic and Racial Minorities & Socioeconomic Status. https://www.apa.org/pi/ses/resources/publications/minorities#:~:text=These%20communities%20commonly%20share%20characteristics,these%20problems%20that%20plague%20communities. Accessed 20 June 2024.

[90] GOV.UK. "Population of England and Wales." Office for National Statistics. https://www.ethnicity-facts-figures.service.gov.uk/uk-population-by-ethnicity/national-and-regional-populations/population-of-england-and-wales/latest (accessed 11th of August, 2023)

[91] GOV.UK. "Regional ethnic diversity." Office for National Statistics. https://www.ethnicity-facts-figures.service.gov.uk/uk-population-by-ethnicity/national-and-regional-populations/regional-ethnic-diversity/latest (accessed 11th of August, 2023)

